# Automated phenotyping of *Caenorhabditis elegans* embryos with a high-throughput-screening microfluidic platform

**DOI:** 10.1038/s41378-020-0132-8

**Published:** 2020-04-06

**Authors:** Huseyin Baris Atakan, Tunc Alkanat, Matteo Cornaglia, Raphaël Trouillon, Martin A. M. Gijs

**Affiliations:** 1 Laboratory of Microsystems, Ecole Polytechnique Fédérale de Lausanne, 1015 Lausanne, Switzerland; 2 Department of Electrical Engineering, Eindhoven University of Technology, 5600MB Eindhoven, The Netherlands

**Keywords:** Engineering, Chemistry

## Abstract

The nematode *Caenorhabditis elegans* has been extensively used as a model multicellular organism to study the influence of osmotic stress conditions and the toxicity of chemical compounds on developmental and motility-associated phenotypes. However, the several-day culture of nematodes needed for such studies has caused researchers to explore alternatives. In particular, *C. elegans* embryos, due to their shorter developmental time and immobile nature, could be exploited for this purpose, although usually their harvesting and handling is tedious. Here, we present a multiplexed, high-throughput and automated embryo phenotyping microfluidic approach to observe *C. elegans* embryogenesis after the application of different chemical compounds. After performing experiments with up to 800 embryos per chip and up to 12 h of time-lapsed imaging per embryo, the individual phenotypic developmental data were collected and analyzed through machine learning and image processing approaches. Our proof-of-concept platform indicates developmental lag and the induction of mitochondrial stress in embryos exposed to high doses (200 mM) of glucose and NaCl, while small doses of sucrose and glucose were shown to accelerate development. Overall, our new technique has potential for use in large-scale developmental biology studies and opens new avenues for very rapid high-throughput and high-content screening using *C. elegans* embryos.

## Microfluidics: High-throughput screening of embryos

A new microfluidic approach enables the high-throughput and automated phenotyping of *C. elegans* embryos. Understanding the effect of osmotic stress-induced damage on cells is vital for understanding a number of biological processes. *C. elegans* embryos are considered ideal model systems for osmotic studies, but their phenotyping is traditionally a labor intensive and time-consuming process. Now, a team from EPFL in Switzerland led by Martin Gijs demonstrate a microfluidic platform for high-throughput phenotyping of *C. elegans*. Embryos are exposed to various osmotic compounds, followed by an automated phenotyping script to assess phenotypes via machine learning and image processing. Their proof of concept experiment demonstrates that their technique can be used for a systems-based approach for osmotic studies.

## Introduction

Survival in osmotic conditions and the ability to repair osmotic stress-induced damage are crucial for the long-term maintenance of cellular life^1^. *Caenorhabditis elegans* long ago emerged as a suitable model organism for osmotic studies^2^. These nematodes present several advantages for in-depth studies to increase the understanding of the mechanisms underlying animal cell adjustment to osmotic conditions. Their well-mapped genome, small size, short lifespan and life cycle make them excellent multicellular organisms to address these fundamental biological questions^3^. It has been previously demonstrated that high levels of osmotic changes in the environment induce the formation of mitochondrial reactive oxygen species (ROS)^4^ and the accumulation of osmolytes such as glycerol in *C. elegans*^5,6^. Additionally, hypertonic or hyperosmotic conditions cause the loss of water, which triggers an intracellular ionic imbalance and, hence, protein aggregation^7^. Therefore, insight into the molecular mechanisms of osmotic damage in *C. elegans* may be important for unravelling neurodegenerative diseases^8^ and inflammatory disorders^9^. The characterization of the genetic basis of animal cell osmoregulation by using *C. elegans* can fill major gaps in our molecular understanding of other cell types, such as mammalian cells^5^.

Previous studies involving the application of an osmotic stressor on the nematode *C. elegans* mostly focused on the influence of high chemical compound concentrations on lifespan. Lifespan reduction can be induced by high molarity NaCl^10^, but can be recovered with the addition of sterilizing compounds such as 5-fluorodeoxyuridine. Osmotic effects can also be induced with sucrose, a molecule composed of two monosaccharides, glucose and fructose. In addition to the osmotic influence of NaCl and sucrose, there might be additional toxic effects that cause lifespan reduction^11^. For instance, treatment with sorbitol—an acyclic polyol used as an osmotic stabilizer—can induce an adaptive osmotic response to compensate for the lifespan reduction. Hypertonic conditions induced by NaCl, sucrose and glucose can also reduce the survival rate of nematodes and significantly alter body size and shape^12,13^. Additionally, the deleterious effects of NaCl are greater than those of sucrose at the same molarity^5^. Additional phenotypic results also revealed that high doses of glucose severely affect the motility of nematodes^14^.

Lab-on-a-chip systems provide powerful solutions for bioanalytical applications, and microfluidics technologies have been shown to enable automated studies of many complex biological systems^15,16^. Extensive phenotypic data can be collected during experimentation with uni- or multicellular organisms. A variety of designs and applications for use in investigations of *C. elegans* at the larval or adult stage have been proposed. However, these technologies have been rarely utilized for embryonic studies so far^17^, despite the advantages of embryos, such as their fast developmental time and their relatively immobile nature. It is well established that early life stage events can have a large impact on nematode development and lifespan^18^. Interestingly, nematode morphologies in later life can also be predicted from early-embryo stage variations^19^. Hence, it is worth investigating whether *C. elegans* embryos could potentially be exploited to perform toxicity or osmoticity studies.

Traditionally, embryonic development imaging studies were conducted by mounting embryos on agar pads, which is highly laborious^20^ and does not utilize a constant chemical aspiration system. Recent microfluidic developments have facilitated the handling of *C. elegans* embryos. A microfluidic platform with microwells was used to mount and observe early embryos obtained by dissecting adults^21^. An initial fluidic design for handling *Drosophila melanogaster* embryos was proposed^22^. This design was later adapted, and an automated microfluidic platform with *C. elegans* embryo incubators was created to observe the embryogenesis of various strains^23^. Other researchers investigated drug delivery to embryos obtained from fertile nematodes^24^, while observation of the first cellular division after mechanically compressing adult nematodes for embryo release was demonstrated^25^. However, all these platforms accommodated only a few embryos, functioned under only a single condition and lacked automated phenotyping of embryonic phenotypes.

The nematode *C. elegans* is a well-established model organism in terms of rapid data extraction through automated phenotyping studies^26^. Previously, various tracking methods were applied to nematodes to provide high-content phenotypic data^27–29^. However, very few automated phenotyping methods have been proposed for *C. elegans* embryos. An embryo phenotyping approach that automatically locates and segments cells and nuclei based on video recordings was reported^30^. Alternatively, cell shape analysis was also conducted using image processing with cell membrane segmentation^31^. Another approach showed the possibility of automated cell lineage tracing^32^. During *C. elegans* embryogenesis, cell movement, division, and death were automatically detected. However, no platform so far has provided (i) an assessment of the physiological state (alive or dead) of the embryo and (ii) the detection of the embryogenesis stage with a high-throughput, systematic and fully automated method.

In this work, we present a high-throughput multiplexed microfluidic platform for automated phenotyping of *C. elegans* embryos during their full development. We used wild-type and mitochondrial unfolded protein response reporter *hsp-6::gfp* embryos to evaluate the stress response. *Caenorhabditis elegans* embryos were obtained through a classical bleaching procedure in gravid adult animals for rapid and massive embryo extraction. The developmental influence of embryo exposure to various osmotic compounds and the eventual after-effects of the potentially toxic bleaching procedure, resulting in embryo mortality, was studied on-chip. Our automated phenotyping script, which utilizes deep learning and image processing, produced accurate results in a relatively short amount of time that, in most cases, matched the results obtained by a human operator. We believe this is the first report of a massive embryo handling protocol combining high-throughput and multiplexed observations with automated phenotyping. The severe developmental lag and lethality caused by high molarity glucose and NaCl in embryos was observed, and the results were in agreement with those obtained in prior works on adult worms. Overall, our phenotypic embryo developmental results indicate that *C. elegans* embryos may represent an alternative to adult worms and lead to comparable experimental conclusions over shorter time periods.

## Results

### Microfluidic chip design

**Fig. 1:**
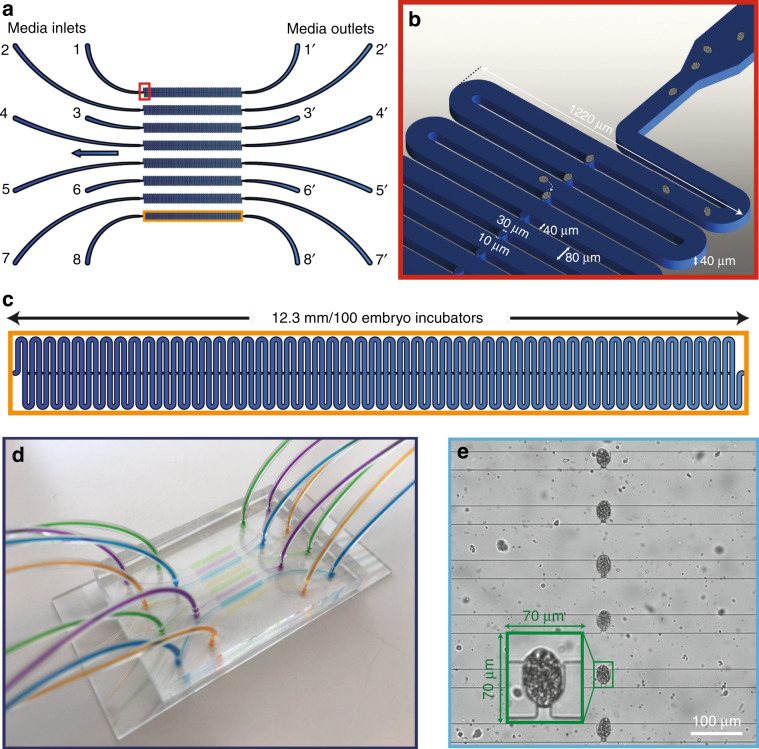
Details of the high-throughput microfluidic platform used for automated phenotyping of *C. elegans* embryos. **a** Schematic representation of the microfluidic chip with eight multiplexed lanes with one end connected to the media inlet and the other to the outlet. **b** Zoomed-in image of the entrance of a microfluidic lane with the feature sizes marked. **c** Image of a single lane that consists of 100 embryo incubators within 12.3 mm. **d** A picture of the PDMS chip (35 × 50 mm) filled with dye solutions and bonded onto a standard glass microscope slide (38 mm × 75 mm). **e** An image of six embryo incubators visualized during time-lapse imaging; a single incubator containing an embryo (in green) is displayed that was utilized by the automated script for phenotyping

The design and technology allows a massive amount of *C. elegans* embryo observation at high resolution and permits highly parallel studies. The semi-automated fluidic pipeline also allows the dramatic reduction of the influence of the external operator on the experiments. Our device is solely based on passive hydrodynamics without integration of any active on-chip components, resulting in a robust and user-friendly device. The design consists of eight side-by-side microfluidic lanes that impose up to eight different conditions on embryos in parallel (Fig. 1a). Each lane has a media inlet that is connected to either an S-medium or waste reservoir and a media outlet that is connected to either an embryo suspension or chemical solution. During the initial microfluidic chip filling and degassing, S-medium was dispensed from the media inlet towards the media outlet by means of a syringe pump. Otherwise, media outlets were used to draw either an embryo suspension or a chemical solution towards the media inlet, which was linked to a media reservoir (see “Materials and methods” for details). We based our design of the embryo incubators on a previously proposed platform^23^, which we configured with slightly larger (10 µm) embryo incubator gates (Fig. 1b). This was found to reduce clogging due to worm debris generated by the bleaching procedure and hence increase the probability of correctly filling the embryo incubator with a *C. elegans* embryo. All worm culture chambers and other redundant components from the previous design were removed to create a rapid embryogenesis observation platform. Each microfluidic lane consists of 100 concatenated embryo incubators so that up to 100 embryos can be observed under a single condition (Fig. 1c). We used standard polydimethylsiloxane (PDMS) fabrication processes to create our microfluidic chips by plasma sealing the PDMS part containing the microfluidic channel design to a standard glass microscope slide (Fig. 1d). Throughout our studies, we recorded images of six embryo incubators at once to save data space (Fig. 1e). Unlike previous ×63 oil immersion brightfield^33,34^ or differential interference contrast imaging^35,36^ of *C. elegans* embryos, we found that ×20 brightfield imaging was sufficient for our case studies. To further decrease the automated phenotyping time, we cropped 70 µm × 70 µm image patches around the embryo incubators and performed our image processing algorithm on these patches.

### Platform working principle

We designed a robust and repeatable experimental protocol to rapidly load *C. elegans* embryos into the incubators and initiate developmental monitoring. We aimed to reduce the operator’s influence on the experiment to a minimal level; therefore, identical serial port commands for the syringe pump were used during the initialization of each experiment to set the same flow rates and injection amounts.

**Fig. 2:**
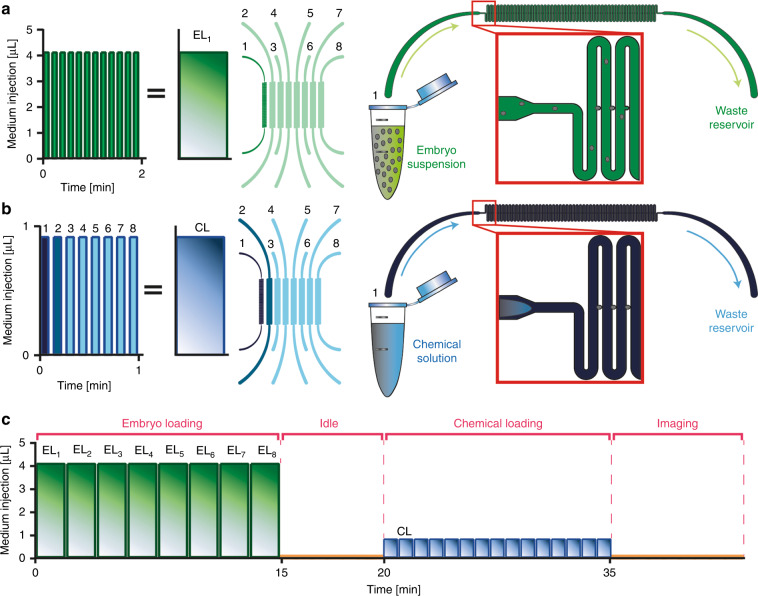
Operation modes of the microfluidic chip. **a** In the basic embryo loading step (denoted as EL), a 150 µL embryo suspension in S-medium was injected from the media outlet into the media inlet for 2 min for each microfluidic lane, thereby filling almost all embryo incubators in that lane with *C. elegans* embryos. **b** In the basic chemical loading step (denoted as CL), all microfluidic lanes were filled with the chemical solution of interest using a directed flow from the media outlet into the inlet for 1 min for eight microfluidic lanes. **c** The overall experimental protocol consisted of basic embryo loading steps of ~15 min, which were used to fill 800 embryo incubators with embryos, a step of 5 min of idle time for focus re-adjustments, basic chemical loading steps of ~15 min for chemical solution loading and 12 h of imaging

In the experimental initialization protocol, the main steps are the embryo and chemical loadings. Eppendorf reservoirs containing a dense population of *C. elegans* embryos suspended in 150 µL filtered S-medium were plugged into the media outlets (Fig. 2a). We started loading embryos after the initial microfluidic chip filling and degassing steps. By passive fluidic dynamics, each embryo found its way to one of the embryo incubators at a flow rate of 625 nL/s. The liquid velocity profile and pressure distribution within embryo incubators were simulated to observe the influence of hydrodynamics on incubator filling (see Fig. S1). From time to time, debris resulting from the bleaching step entered some embryo incubators, but the high number of incubators per lane compensated for this loss. If the embryo suspensions were residue free, up to 100 incubators could be filled in <2 min in each microfluidic lane. The embryo loading step, which was performed at a flow rate of 625 nL/s, yielded 84% embryo incubator occupancy for the entire experiment. Following embryo loading, the embryo suspension reservoirs were replaced with reservoirs of 600 µL chemical solution (Fig. 2b). These solutions contained the chemical compound of interest at a certain concentration. The microfluidic lanes were fully filled with the solution in an alternating manner with a flow rate of 156 nL/s, as presented in Fig. 2b, c. We divided the total chemical injection amount over 15 cycles. After completing the first chemical loading cycle for one microfluidic lane, the injection in the neighboring lane was started. Once the first cycle for all eight lanes was completed, the next cycle was started with a chemical injection in the initial microfluidic lane. The goal was to synchronize the chemical exposure time of the different embryos in the parallel microfluidic lanes. In between the embryo and chemical loading steps, the focal planes were adjusted (∼5 min), as the reservoir plug-in/plug-out steps caused a focal plane shift (idle time in Fig. 2c). Under ideal conditions, we could fill up to 800 embryo incubators in 15 min (see the example of the device operation during an experiment in Fig. S2). The total experimental preparation time could be reduced to ∼35 min. Our easy-to-use and semi-automated platform could therefore set up a large number of *C. elegans* embryos for developmental studies in an extremely short amount of time. Our fluidic protocol was finalized by maintaining a gentle 42 nL/s flow to maintain the embryos in the incubators for 12 h of time-lapsed imaging. This flow was required to retain the embryos inside the incubators.

### Automated detection of embryonic states and embryogenesis stage transitions

We developed an automated embryo stage detection and fluorescent signal quantification algorithm for rapid, high-content phenotyping of *C. elegans* embryos with MATLAB (MathWorks, Natick, MA, USA). We processed brightfield and fluorescent images that were acquired during the 12-h time-lapsed imaging, which was performed at 5-min intervals for embryogenesis and 10-min intervals for the mitochondrial stress expression studies. The automated embryo development analysis algorithm consisted of three main parts: (i) detection of the locations of the different embryo incubators, (ii) analysis of the embryo states, and (iii) detection of the stage transitions during embryogenesis. Detection of the embryo incubator locations took place once for each relevant position on the motorized microscope stage, whereby each physical position provided an image of a unique set of incubators on the microfluidic chip with a Canny edge detector (see further details in Fig. S3)^37^. After revealing the *x*- and *y*-coordinates of each embryo incubator at each position on the motorized stage, we cropped 70 µm × 70 µm image patches (corresponding to 200 × 200 pixels) around each embryo incubator to be used for the following parts of our algorithm.

After preliminary observations of embryogenesis, we established the classification of different embryo developmental stages. *Caenorhabditis elegans* embryos pass through several embryogenesis stages, and we focused on detecting some of the most significant ones, namely, the bean, twitching, and hatching stages^23^ (Fig. S4). During the experiments, some embryos were dead, some embryos had visually unclear bean phases (because they had already passed the bean stage or due to embryo orientation in the incubator), some embryos did not hatch within 12 h, and some embryos developed normally and showed clear bean, twitching, and hatching stages. Additionally, some embryo incubators were empty during the course of the experiments because of debris accumulation or a low embryo concentration in the suspension. The above-mentioned states were named “Dead,” “Unclear Stage,” “Late Hatching,” “Normal,” and “Empty Embryo Incubator.” Our goal was simply to classify the experimental image patches in these different categories.

**Fig. 3:**
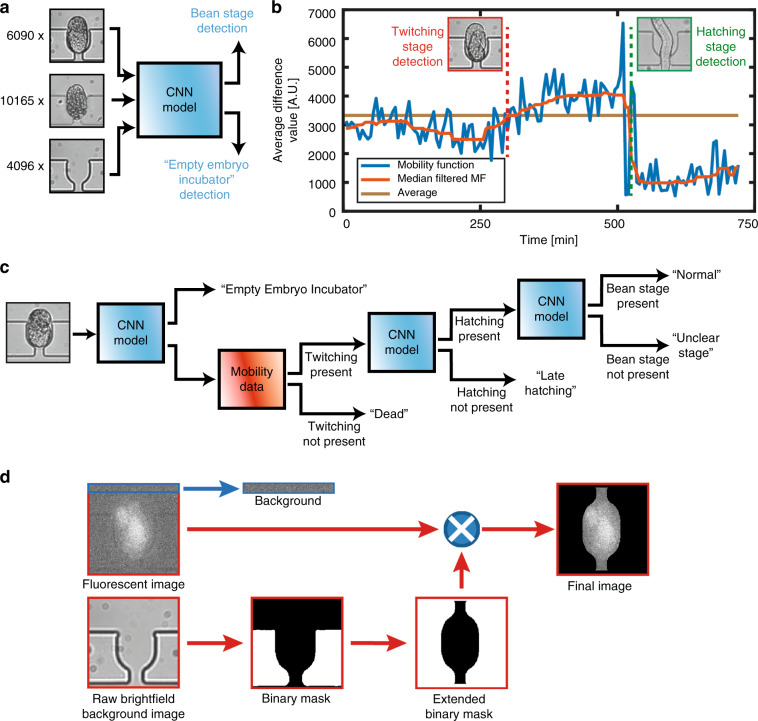
Automated image analysis for the extraction of *C. elegans* embryo phenotypes and fluorescent signal expression. **a** An initial training data set of 6090 prelabelled embryos in the bean stage, 10165 prelabelled embryos prior to the bean stage, and 4096 images prelabelled as “Empty Embryo Incubator” were provided. **b** The difference between the averaged pixel values of two consecutive 70 µm × 70 µm embryo image patches in a time-lapsed sequence (“Average Difference Value,” also named “mobility data”) was used as an indicator to detect an instance of the twitching transition. **c** Block diagram of the protocol. To detect an “Empty Embryo Incubator,” the hatching stage transition and the bean stage transition, we used the convolutional neural network model (see Fig. S11). The mobility data were used to determine whether an embryo was alive or “Dead.” **d** For the fluorescent signal analysis, a binary mask was created, extended and mapped onto the fluorescent image to target only the background-free embryo. The median intensity value inside the final image was calculated to compute the embryo’s fluorescent intensity. This result was corrected using a 70 µm × 7 µm background window taken from the top part of an image patch to take into account inevitable variations in lamp intensity

We employed a combination of convolutional neural network (CNN) and standard image processing techniques to detect the above-mentioned three embryogenesis stage transitions and the five embryo or incubator states (see details in “Materials and methods”). For the training of the simplified AlexNet architecture^38^—which made use of features extracted using CNN—we provided three types of image patches corresponding to the “Empty Embryo Incubator” (4096 images), “Pre-bean Stage” (10165 images), and “Bean Stage” (6090 images) states (Fig. 3a). In parallel, we benefited from the mean temporal information regarding the pixel intensities in the image patches. Following the pixel-by-pixel subtraction of two consecutive image patches, we determined the average of these differences, which we considered according to the mobility function of the embryo under consideration (see Fig. 3b for the mobility data for a “Normal” embryo). We utilized a sequence of steps utilizing the CNN model and the mobility function to extract the phenotypic parameters (see the block diagram in Fig. 3c) and provided a few typical experimental examples of classification in Fig. S5. We developed a standard image processing approach to quantify the fluorescent signal expression in embryos (Fig. 3d; further details are given in the “Materials and methods”).

### Osmotic influence on embryo development

**Fig. 4:**
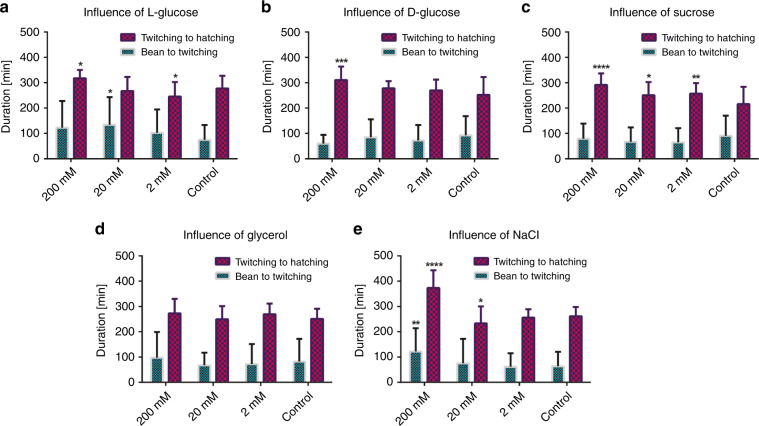
Study of the influence of various chemical compounds on the development time of “Normal” wild-type *C. elegans* embryos. Influence of **a** l-glucose, **b** d-glucose, **c** sucrose, **d** glycerol, and **e** NaCl on the bean-to-twitching and twitching-to-hatching time intervals, respectively. Data are expressed as the mean ± SEM, *p ≤ 0.05, **p ≤ 0.01, ***p ≤ 0.001, and ****p ≤ 0.0001. All measurements were based on *N* = 15–65 embryos

We studied the influence of various compounds and osmotic conditions that were previously reported to be significant on the lifespan, survival, life expectancy, body shape, and stress induction of *C. elegans* worms. Our goal here was to investigate the suitability of *C. elegans* embryos for several case studies previously performed in adult nematodes. For this purpose, we exposed wild-type embryos to 200, 20, and 2 mM concentrations of l-glucose, d-glucose, sucrose, glycerol, and NaCl and characterized the influence of these compounds on embryo development and survival at room temperature with our automated script (Fig. 4 and Table 1). We considered only “Normal” embryos for our analysis to standardize the experimental set of embryos, for which we could clearly observe development prior to the bean stage (Fig. 4). We observed a variation in the embryo lethality rate in the control conditions in the different chemical studies (Table 1). Even though there was identical media exposure (filtered S-medium with no other chemicals) in the different experiments, such variations might result from the differences in the adult worm population present on the plate (see “Materials and methods”), the local *Escherichia coli* distribution profile on the plate, which might contain slightly advanced or developmentally lagging worms, minor variations in the exact bleaching duration, and operator-based influences during bleaching. During the bleaching procedure, we regularly checked the embryo suspension, and if all the adult body fragments had disappeared, we centrifuged the solution. Otherwise, any remaining adult body fragments would likely clog the microfluidic lanes and incubators. We performed an off-chip characterization of the embryo suspension one day after egg harvesting, and the resulting data is shown in Fig. S6, indicating a dead embryo percentage variation of 20–60% after the bleaching procedure. This experiment proved that variability in embryo development can be due to hazardous effects that are a consequence of the toxic bleaching procedure. We did not notice such a high mortality rate in our prior work, which relied on the parental generation to lay embryos, which therefore did not result in a rapid assay^23^.

**Table 1:** The fraction of “Dead” embryos over all embryos and of the “Normal” embryos over the alive embryo population results are illustrated for 200 mM, 20 mM, 2 mM, and control (0 mM) conditions of l-glucose, d-glucose, sucrose, glycerol, and NaCl for wild-type *C. elegans* embryos

Compound	Dose	Dead embryo (%)	Normal embryo over alive embryo (%)
l-Glucose	200 mM	59.8	29.7
	20 mM	36.3	28.5
	2 mM	22.2	34.3
	Control	38.5	46.7
d-Glucose	200 mM	56.1	20.8
	20 mM	52.7	47.9
	2 mM	37.2	56.5
	Control	48.0	47.2
Sucrose	200 mM	19.4	35.9
	20 mM	25.7	36.2
	2 mM	27.4	31.5
	Control	30.1	33.6
Glycerol	200 mM	37.1	37.1
	20 mM	29.0	30.7
	2 mM	44.9	32.6
	Control	36.5	23.5
NaCl	200 mM	42.8	22.3
	20 mM	32.6	38.7
	2 mM	29.3	34.9
	Control	18.5	43.3

d-Glucose, which is usually referred to as glucose, is an important energy source, and l-glucose is the laboratory-synthesized enantiomer. l-Glucose is not biologically active, and this compound affects the nematode differently. Previous studies determined the different impacts of these two enantiomer compounds on lifespan^39^, motility^14^, the egg laying rate^40^, and nematode sensitivity^41^.

We observed a developmental time increase, especially for the twitching-to-hatching duration, in the presence of high molarity l-glucose (Fig. 4a). More specifically, at a 200 mM concentration, the average bean-to-twitching interval was 48 min, and the twitching-to-hatching interval was 40 min more than that in the control condition. This was also accompanied by a 55% increase in the “Dead” embryo percentage and a 36% decrease in the “Normal” embryo percentage compared to that of live embryos, which was the sum of the “Normal,” “Late Hatching,” and “Unclear” populations (Table 1). This means that extreme doses of l-glucose were hazardous, as they increased both the development time and the “Dead” embryo rate and reduced the successful detection of “Normal” embryos. Interestingly, a similar developmental time profile was also observed for d-glucose (Fig. 4b), which had a more severe impact on the survival of the embryos. The twitching-to-hatching development interval was increased by 60 min compared to that of the control at a 200 mM concentration. A 17% and 10% increase in the “Dead” embryo percentage was also noticed at the 200 and 20 mM concentrations, respectively (Table 1). We also observed a severe drop (∼55%) in the “Normal” embryo percentage only for the highest concentration of glucose. Embryos treated with 200 mM d-glucose were problematic for bean stage detection due to embryo deformation caused by this dose. l-Glucose cannot be used by living organisms as a source of energy, although it showed a similar trend to that of d-glucose. The effect of these enantiomers on embryos was therefore expected to be mostly osmotic rather than arising from specific biochemical mechanisms. However, the 47.9% and 56.5% percentages of “Normal” embryos observed at the 20 and 2 mM d-glucose concentrations, respectively, may indicate a limited effect at these concentrations, with toxicity being more prevalent at more extreme doses.

Sucrose, a molecule composed of glucose and fructose, was utilized to create hypertonic conditions for *C. elegans*. Previous studies demonstrated a decreasing trend in nematode survival caused by a rise in the osmotic stress caused by an increase in the sucrose dose^5,12^. We found similar results for the influence of the sucrose dose on *C. elegans* embryos compared to that on adult nematodes. We detected an almost linear developmental time increase along with an increase in the molarity (Fig. 4c). Indeed, the average twitching-to-hatching time interval increased from 216 min (for the control condition) to 292 min (for 200 mM sucrose) in a mostly linear fashion on a log scale. Nonetheless, a strong effect, which was reflected as a decline in the “Dead” embryo percentage as the molarity increased, was noted (Table 1). The lethality rate dropped by 36%, 15% and 9% at the 200 mM, 20 mM, and 2 mM concentrations, respectively. There was no influence of sucrose molarity on the detection of “Normal” embryos. We concluded that there was a less lethal outcome for embryos exposed to sucrose versus glucose.

Previous studies with two types of glucose and sucrose revealed that the influence of these compounds could be due to either a toxic effect or an osmotic effect. The main influence of these chemical compounds on embryo development has not yet been established. For this reason, we performed an osmotic study on embryos using logarithmic dilutions of the nontoxic polyol compound glycerol. Prior research with glycerol^39^ reported a lifespan reduction in nematodes and an adaptive response of the nematode to osmotic glycerol exposure^42^. Our results showed that there was no significant impact of osmotic conditions resulting from glycerol on the development time (Fig. 4d). We noticed a dose-independent variation in the “Dead” embryo percentage and, interestingly, a beneficial influence on the detection of “Normal” embryos at high molarity (Table 1). The “Normal” embryo detection percentage increased within the range of 31–58% with the addition of glycerol during embryo development. We established that the nontoxic compound glycerol had a different outcome on embryo development than on nematode development.

NaCl is known to be a highly toxic and ionic component that severely affects the nematode *C. elegans*, although nematodes show tolerance to a certain extent^43^. NaCl has been demonstrated to have a dramatic impact on the survival^1,44^, lifespan^10,11^, aging^13^, and body volume^5^ of the nematode. We observed the effects of a highly toxic dose on the development time at a 200 mM concentration (Fig. 4e). The twitching-to-hatching development time was almost 50% greater than that observed in any other condition; even the bean-to-twitching time was ~60% greater at this dose than at any other dose. Another toxic indication was the “Dead” embryo rate (Table 1). We noticed that dose-dependent effects, such as the lethality percentage, increased by 58%, 76%, and 131% for NaCl concentrations of 2 mM, 20 mM and 200 mM, respectively. In addition, the “Normal” embryo percentage was greatly influenced at the 200 mM dose. It dropped by almost 50% compared to that detected in the control condition. These findings were correlated with previous research on *C. elegans* embryo survival under hypertonic conditions resulting from NaCl^45^. We speculate that such a strong influence of NaCl compared to that of other compounds might also be due to the 2-fold increase in the osmolarity generated due to the dissociation of the NaCl molecules.

We additionally investigated the power and accuracy of our phenotyping algorithm in each osmotic study visually. We classified embryo states and embryogenesis stage transitions for all embryos. Our results demonstrated a close link between the manually classified states and the CNN and mobility function script results (Figs. S7, S8 and Table S1). We noticed an overall decrease in the correlation between the manually classified and automated script-based detection results for the bean stage (in the range of 51–64%; see details in Tables S2–S7). Additionally, we noticed a similar decrease at certain chemical concentrations in bean stage detection. The main explanation for this decrease was the zero tolerance of manual and automatic classification. This meant that the manual classification of a stage transition by observing two consecutive images in a time-lapse sequence must be the same as that obtained with the automated classification; in other words, the stage change should be detected in exactly the same frame of the time-lapse sequence. In the same supporting tables, we also shared our results after including a 5-frame tolerance range. This approach significantly increased the correlation between the results of the manual classification and automated script-based detection. In the future, bean stage detection could be improved by using more and clearer bean stage images in the CNN training data.

We created a clustergram profile from the normalized mean values of our measurement results (see Fig. S9). Such a clustergram approach provides a normalized comparison of the various chemicals and eliminates intra-control variations, which leads to an assessment of the most important phenotypes that highlight the influence of a chemical on embryo viability. We concluded that the most crucial phenotype percentages for evaluating embryo viability were the “Normal” and “Dead” embryo population percentages. Interestingly, we noticed that a healthy embryo development indicator was the shortening of the bean-to-twitching stage duration. The clustergram was demonstrated to be a useful method to illustrate all data in a graphical fashion by highlighting the key phenotypes and the most influential chemicals for embryo development.

### Mitochondrial stress induction under osmotic stress

Osmotic stress causes a loss of water, which leads to an intracellular ionic imbalance and protein aggregation in the nematode^8^. It is also the origin of the formation of methylglyoxal-modified mitochondrial proteins and ROS^4^. Accordingly, osmotic conditions likely alter the function of mitochondria in nematodes. Therefore, we focused on the mitochondrial behavior of *C. elegans* embryos under osmotic conditions. We analyzed the development of the mitochondrial unfolded protein response (UPR^mt^) reporter strain *hsp-6::gfp*^46^ and evaluated the induction of the UPR^mt^ under hyperosmotic environmental conditions. This reporter, which is associated with mitochondrial function, is considered a key factor in aging. The UPR^mt^ is one of the stress responses deployed by cells to maintain mitochondrial function^47^.

**Fig. 5:**
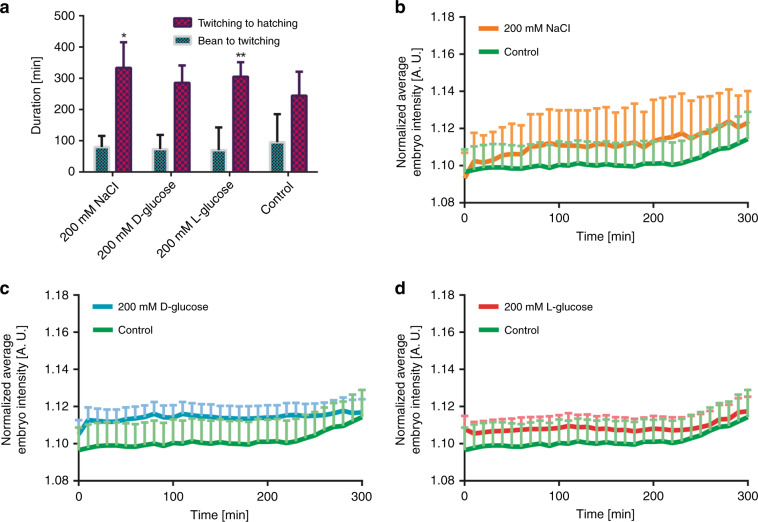
Study of the effect of 200 mM of various chemical compounds on the development of “Normal” *hsp-6::gfpC. elegans* embryos. **a** Influence of NaCl, d-glucose, and l-glucose on the bean-to-twitching and twitching-to-hatching time intervals. Mitochondrial stress induction in embryos was shown by the background-corrected fluorescence during the initial 300 min of embryogenesis for **b** 200 mM NaCl, **c** 200 mM d-glucose, and **d** 200 mM l-glucose compared to the control condition. Data are expressed as the mean ± SEM, *p ≤ 0.05 and **p ≤ 0.01. All measurements were based on *N* = 6–27 embryos

We used the three most influential compounds at 200 mM concentrations (l-glucose, d-glucose, and NaCl) based on our developmental study of wild-type embryos. We analyzed the developmental behavior of “Normal” *hsp-6::gfp* embryos under hyperosmotic conditions (Fig. 5a). All three compounds were a source of a lag in the development time, as already observed in the wild-type embryo study. The highest impact resulted from ionic NaCl at 200 mM. The average twitching-to-hatching development time for NaCl was increased by 89 mins compared to that of the control condition. We noticed an inverse profile for the bean-to-twitching stage (*p* values > 0.05) compared to that of the twitching-to-hatching stage. The main reason for such behavior is the minimal error tolerance due to automated phenotyping with 10-min intervals and the relatively short development duration of the bean-to-twitching stage compared to that of the twitching-to-hatching stage (which was more error-tolerant due to an increase in the development time). This was confirmed after obtaining the manually classified data (see Fig. S8). The “Dead” embryo percentage was elevated in all osmotic conditions, and similarly, NaCl produced the highest lethality rate with a 72% increase compared to that of the control condition (Table 2). We noticed a 50% drop in the “Normal” embryo percentage for 200 mM NaCl. However, this influence was not very apparent for the two glucose types. We observed a decline in the accuracy of “Dead” embryo classification in the presence of 200 mM NaCl, which was similar to that observed in the wild-type embryo study (see the accuracy results in Table S7).

**Table 2:** The fraction of “Dead” embryos over all embryos and of “Normal” embryos over the alive embryo population results are illustrated for 200 mM NaCl, 200 mM d-glucose, 200 mM l-glucose, and the control condition (0 mM) of *hsp-6::gfp* *C. elegans* embryos

Compound	Dose (mM)	Dead embryo (%)	Normal embryo over alive embryo (%)
NaCl	200 mM	77.1	18.8
d-Glucose	200 mM	51.6	35.5
l-Glucose	200 mM	52.4	33.9
Control	0 mM	44.7	37.0

For the “Normal” embryos, we investigated the population-averaged time-dependent UPR^mt^ expression profile (Fig. 5b–d). We focused on the initial 300 min of the experiment, as most of the embryos were present in the incubator in this time interval. The data corresponding to the entire duration of the experiment can be found in Fig. S10. After 300 min, we noticed an increase in the intensity profile in parallel with the advancement of embryonic development, as shown in Fig. 5b–d.

The *hsp-6::gfp* embryos were previously demonstrated to generate a rather weak fluorescent signal^23^. We noticed that the basal intensity expressed by an average embryo under the control condition was ~1.1–1.2 AU (see Fig. 5b–d) because of the development of the weakly fluorescent larva inside the eggshell. Exposure to various chemical compounds at 200 mM exhibited an insignificant >1-fold increase in induction (*p* value > 0.05), while it is known that impaired mitochondrial respiration increases UPR^mt^ signal expression as an indicator of a stress response to promote lifespan^48,49^. We hypothesize that the consistently insignificant UPR^mt^ increase in embryos along with the extended development time (Fig. 5a) at 200 mM concentrations of NaCl, l-glucose, and d-glucose may be an indicator of slightly compromised mitochondria. Owing to our technique, we were able to capture the fluorescent dynamics of *C. elegans* embryos in combination with the viability phenotypes.

## Discussion

In this work, we presented a new multiplexed and automated phenotyping microfluidic platform for high-throughput *C. elegans* embryogenesis studies. To the best of our knowledge, no other platform has demonstrated such a high level of throughput combined with parallel and automated experimental protocols for embryogenesis studies. We configured each microfluidic lane for different parallel conditions to simultaneously observe the influence of various compounds on embryonic development. Owing to the integration of microfluidics, we continuously perfused well-characterized doses of compounds into the microchannels, guaranteeing a level of experimental control that is unattainable for the traditional methods using agar plates. We can incubate up to 100 embryos in a single microfluidic lane, extract five phenotypes for the incubator occupancy, and detect the three important life stages of embryogenesis. We observed the experiment for up to 12 h after the initial embryo placement in an automated manner. We investigated, on average, 147 “Normal” embryos in the assays for each experiment, thereby indicating that our microfluidic platform can be used for medium-to-high-throughput studies.

We intentionally designed our microfluidic platform to contain only simple, passive components, and we used standard soft-lithographic fabrication procedures. The simplicity of the design and the user-friendliness of the semi-automated protocol are aimed at researchers beyond the microsystem community and including biologists who are active in nematode research. Traditional ×63 oil immersion imaging was replaced by ×20 imaging in our protocol to reduce computational time and data storage space, but ×63 imaging can still be effortlessly incorporated into the system.

We created repeatable serial port commands for the syringe pump, which generated a simple experimental preparation pipeline and reduced laborious operator involvement. The initial setup time of the experiment was reduced to 35 min. Additionally, the experiment demanded no active involvement of the user once it was initiated, in sharp contrast with embryo studies performed on agar plates. A significant amount of data was efficiently collected in a short time while still providing high-content datasets.

As a proof of concept, we performed a case study of the development of *C. elegans* embryos under osmotic conditions. We loaded several chemical compounds that are known to induce hyperosmotic conditions and that are toxic to adult nematodes. We noticed the high toxicity for wild-type embryos of all NaCl concentrations and the 200 mM concentrations of l-glucose and d-glucose, which resulted in an increased dead embryo percentage and development time. A favorable effect on embryo health was observed with 2, 20, and 200 mM concentrations of sucrose, 20 mM glycerol, and a 2 mM d-glucose concentration, which resulted in an increased number of healthy embryos. NaCl at a 200 mM concentration produced the greatest variation in the phenotypic properties by comparison and had a dominant effect. This phenomenon was understood as being a consequence of the higher osmolarity of the buffer, which led to an osmotic gradient across the eggshell, as the outer embryo layers were permeabilized by the bleaching.

We also demonstrated how embryos attempt to adjust to osmotic environments. We performed an embryogenesis study with the *hsp-6::gfp* worm strain, which exhibits mitochondrial stress in the presence of 200 mM NaCl, d-glucose, and l-glucose. Our results revealed that UPR^mt^ expression and hence mitochondrial function were slightly increased, although this was not statistically significant. Furthermore, the extreme molarities of these compounds resulted in a higher killing rate (in the range of 15–72%) of embryos with respect to that of the control condition. Specifically, 200 mM NaCl caused extremely high lethality.

Beyond the technical advancements of the work, this study supports the use of *C. elegans* embryos in addition to adult worms under osmotic conditions, as they have demonstrated clear responsivity to such assays. The short development time of the embryos and their similar behaviors compared to those observed in adult nematodes under osmotic conditions opens new experimental avenues for additional case studies. We therefore believe that our new microfluidic approach will pave the way for new research methods utilizing embryos as a new model for biochemical and developmental investigations.

## Materials and methods

### Materials and chemicals

Four-inch, 550-µm-thick Si wafers were obtained from the Center of Micro- and Nanotechnology of EPFL (Lausanne, Switzerland). MicroChem SU8-3025 (1L) negative photoresist was purchased from Micro Resist Technology GmbH (Berlin, Germany). PDMS Sylgard 184 was purchased from Dow Corning (Wiesbaden, Germany). Corning microscope slides (75 mm × 38 mm) were purchased from Sigma-Aldrich (Buchs, Switzerland). Microline ethyl vinyl acetate tubes with 0.51 mm inner and 1.52 mm outer diameters were purchased from Fisher Scientific (Wohlen, Switzerland). l-Broth bacterial culture medium was obtained by adding 10 g of Bacto-tryptone, 5 g of Bacto-yeast, and 5 g of NaCl to 1 L of H_2_O. S-basal medium was obtained by adding 5.85 g of NaCl, 1 g of K_2_HPO_4_, 6 g of KH_2_PO_4_, and 1 mL of cholesterol (5 mg/mL in ethanol) to 1 L of H_2_O. S-medium was obtained by adding 0.5 mL of 1 M potassium citrate (pH 6), 0.5 mL of trace metal solution, 0.15 mL of 1 M CaCl_2_, and 0.15 mL of 1 M MgSO_4_ to 50 mL of S-basal medium. The S-basal, l-broth, and S-media were sterilized by autoclaving. The bleach solution was prepared by combining 0.33 mL of 4 M sodium hydroxide, 3.66 mL of deionized water, and 1 mL of 7–10% sodium hypochlorite solution.

### Worm and bacterial culture and embryo extraction

A single colony of *E. coli* strain OP50 was harvested from a streaked plate and injected into l-broth medium. The injected cultures were shaken at 37 °C overnight. The l-broth medium was removed after overnight culturing by centrifugation. Freshly prepared and filtered S-medium was added, and the suspension was vortexed to obtain a uniform bacterial distribution. Nematode growth medium (NGM) plates were then seeded with the *E. coli* OP50 food source for *C. elegans* and cultured at 20 °C. A synchronized population of ~500–600 L1 stage worms was distributed on a seeded NGM plate. Four NGM plates were prepared with the same protocol. After 2 days, when the population reached the adult stage and embryos were observed on the plates, the NGM plates were bleached to extract the embryos (see Fig. S6). We employed bleaching to rapidly collect the eggs, whereas alternative techniques that rely on passive egg collection from mothers that avoid bleaching are also used depending on the interests and goals of the user. While the majority of the embryo population was used during the experiment, some embryos were saved to culture new NGM plates. N2 wild-type and SJ4100 (*hsp-6::gfp*) strains were used and obtained from the Caenorhabditis Genetics Center (University of Minnesota).

### Fabrication of the microfluidic chip

We fabricated our microfluidic chips by soft lithography using single layer SU8 photoresist molds. Conventional photolithography was utilized to deposit a 40-µm-thick layer of SU8 on 4-in. wafers. The SU8 mold was treated with trimethylchlorosilane in a vacuum chamber for 15 min to prevent the adhesion of PDMS during molding. A liquid PDMS mixture (base-to-curing agent ratio 10:1) was poured on the SU8 mold, degassed, and cured at 80 °C for 2 h. Once the PDMS mixture was cured, we removed the device from the SU8 mold and punched 1.5 mm inlets and outlets using a biopsy punch. Both the PDMS device (∼35 mm × 50 mm in size) and the 38 mm × 75 mm glass slide were plasma activated and sealed together. After placing the bonded microfluidic chip on a hotplate at 80 °C for 10 min to enhance the bonding, the chip was mounted on our experimental setup. We used standard PDMS fabrication processes to create our microfluidic chips.

### Experimental preparation

A 12-port rotary valve was connected to a Kloehn syringe pump and coupled to a microfluidic device. The microfluidic device was mounted on a microscopy control system (Visitron, Puchheim, Germany). The microfluidic chip was mounted on the motorized stage, and the eight inlets were connected to the syringe pump and the eight outlets were interchangeably connected to the embryo suspension and chemical solution. Each embryo suspension contained 750–1000 embryos. The two additional ports of the 12-port valve were linked to the waste and S-medium reservoirs. For the embryo and chemical loading, the excess liquid was disposed of in the waste reservoir. A ×20 (0.4 NA) objective was mounted on the setup, and its field of view completely covered the observation area of the six parallel embryos in their incubators. The illumination source alternated between white light for brightfield microscopy and a fluorescence excitation source for fluorescence microscopy. During embryogenesis studies that were performed with a flow rate of 42 nL/s, time-lapsed images of a group of six embryo incubators were recorded every 5 min for the brightfield imaging studies and every 10 min for the dual brightfield fluorescent imaging studies for 12 h.

### Details of the automated detection of the embryo state and embryogenesis stage transition

We made use of features extracted using CNN, which was based on the well-established AlexNet^38^ architecture (Fig. S11a), along with mean temporal difference information derived from the image patches. We simplified the AlexNet architecture for our case study (Fig. S11b). Our algorithm was engaged as follows (Fig. 3c). Initially, our CNN model was utilized to determine if the image patch contained an embryo or if it was embryo free. If an embryo was not present, the embryo incubator under consideration was classified as “Empty.” If it contained an embryo, the algorithm calculated the variance of the mobility function. If the latter was lower than a predetermined threshold, this indicated that the embryo was not in the twitching stage, and the embryo incubator under consideration contained a “Dead” embryo. Otherwise, the twitching stage was detected, and then the embryo under consideration was considered a living embryo in the twitching stage (we classified it as being in the “Normal,” “Unclear Stage,” or “Late Hatching” state). The CNN model was also used to check whether the embryo was in the hatching stage. The hatching stage transition was identified by checking the embryo and detecting the time at which the embryo was no longer present according to the trained CNN. If the hatching stage could not be recorded in the time-lapsed sequence of 12 h, the embryo state was classified as “Late Hatching.” Last, we used the trained network to detect the transition from the pre-bean to the bean stage of the embryo, if it was present. If a clear visual transition could not be determined, then we concluded that we had an embryo in the “Unclear Stage.” Otherwise, we had a “Normal” embryo, where the visual cues of the stage transitions were clear.

We created a reduced mobility function by cropping the median-filtered mobility data to a timeframe that was limited by the time of the hatching stage transition (see Fig. S11c). Our experimental observations revealed that the transition time from the bean stage to the twitching stage could be modeled as an instance where the reduced mobility function intersected with its mean. In the case of a “Late Hatching” embryo, the twitching stage was determined by utilizing the noncropped mobility data.

### Average fluorescent intensity quantification of embryos

We followed a straightforward image processing approach to quantify the green fluorescent protein expression of *hsp-6::gfp C. elegans* embryos. We first created a binary mask from one of the embryo-free patch images, which was extended and mapped onto the fluorescent image (see Fig. 3d). This mask isolated the fluorescent signal of the embryo from the background and took into account only the pixel values originating from the embryo. After the multiplication of these two patches, the irrelevant background was excluded, except for the background openings on the top and bottom of the embryo. However, as the total pixel number of these openings was ~4% of the total pixel number in the unmasked part of the image patch, by simply taking the nonzero median intensity value of this image patch, we obtained a meaningful value for the average fluorescent signal expression. Additionally, a 70 µm × 7 µm background window was taken from the top part of the image patch (see Fig. 3d), and the average fluorescent intensity in this window was utilized to normalize the fluorescent signal intensity expression of an embryo. We determined the corrected value of the average fluorescent intensity of *hsp-6::gfp* embryos with the following formula:

1 $$I_{\mathrm{SBR}} = \frac{{I_{\mathrm{ROI}}}}{{{I}_{{\rm{BG}}}}},$$

where *I*_SBR_, *I*_ROI_, and *I*_BG_ are the normalized average embryo intensity, the average value of the embryo intensity expression, and the average background fluorescent intensity values, respectively.

### Statistical analysis

The developmental duration data were analyzed for statistical significance with one-way analysis of variance (ANOVA) using GraphPad Prism (GraphPad Software, San Diego, CA, USA). The average embryo intensity data were analyzed for statistical significance with repeated-measures one-way ANOVA. To determine the influence of l-glucose on wild-type embryos, we used 19, 33, 48, and 50 embryos for the 200 mM, 20 mM, 2 mM, and control conditions, respectively. To determine the influence of d-glucose on wild-type embryos, we used 15, 34, 65, and 42 embryos for the 200 mM, 20 mM, 2 mM, and control conditions, respectively. To determine the influence of sucrose on wild-type embryos, we used 52, 47, 40, and 39 embryos for the 200 mM, 20 mM, 2 mM, and control conditions, respectively. To determine the influence of glycerol on wild-type embryos, we used 46, 42, 30, and 27 embryos for the 200 mM, 20 mM, 2 mM and control conditions, respectively. To determine the influence of NaCl on the wild-type embryos, we used 23, 48, 37, and 65 embryos for the 200 mM, 20 mM, 2 mM, and control conditions, respectively. To determine the mitochondrial stress induction in *hsp-6::gfp* embryos, we used 6, 27, 20, and 27 embryos for the 200 mM NaCl, 200 mM d-glucose, 200 mM l-glucose, and control conditions, respectively.

## Supplementary information

Movie S1

Movie S2

Movie S3

Supplementary Information

## Data Availability

The data that support the findings of this study are available on request from the corresponding author.
