# Supplementary material for: Automated phenotyping of Caenorhabditis elegans embryos with a high-throughput-screening microfluidic platform
Source: Microsyst Nanoeng. 2020 Apr 6;6:24. doi: 10.1038/s41378-020-0132-8 (PMC8433184; doi:10.1038/s41378-020-0132-8)
Supplement: Supplementary file 4 — Supplementary Information [file 41378_2020_132_MOESM4_ESM.docx]

**Supplementary Information**

**Automated phenotyping of *C. elegans* embryos with a high-throughput-screening microfluidic platform**

**H. B. Atakan^1^, T. Alkanat^2^, M. Cornaglia^1^, R. Trouillon^1^ and M.A.M. Gijs^1^***

^1^Laboratory of Microsystems, Ecole Polytechnique Fédérale de Lausanne, CH-1015 Lausanne, Switzerland

^2^Department of Electrical Engineering, Eindhoven University of Technology, 5600MB, Eindhoven, The Netherlands

*Author to whom correspondence should be addressed.

e-mail: martin.gijs@epfl.ch

**Supplementary Figures**


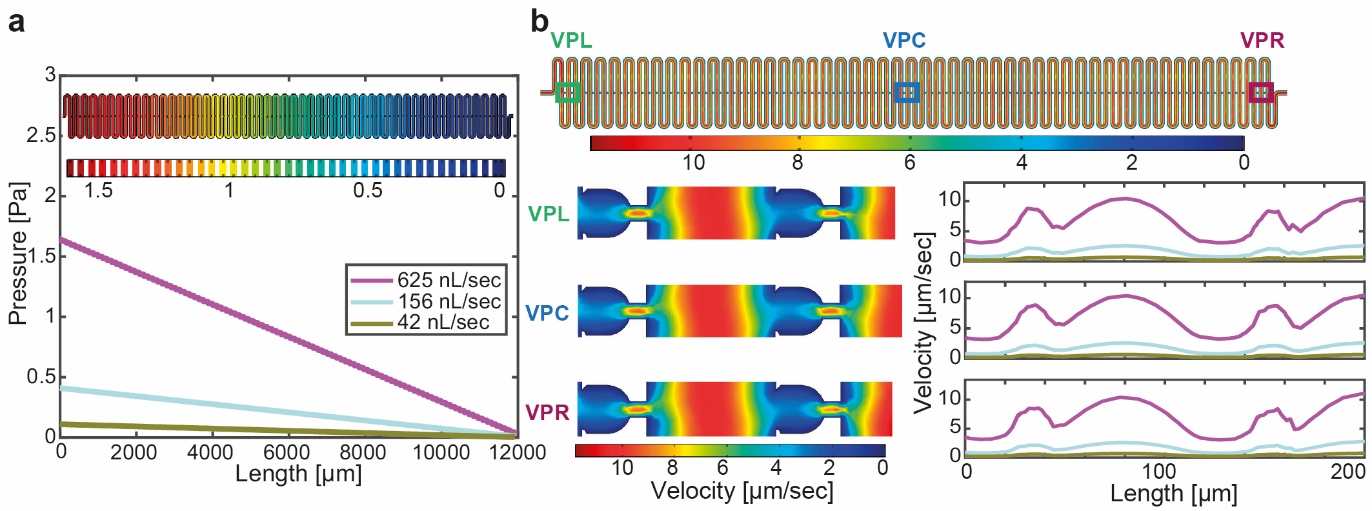


**Fig. S1.** **Pressure and particle velocity simulations along all the embryo incubators in a microfluidic lane. (a)** Pressure distribution profile along a microfluidic lane at three main flow rates used in the experiment (42, 156, 625 nL/sec). The fluidic pressure is adequate to keep embryos in their incubators. **(b)** Liquid velocity profile at the closest point to the media outlet (VPL), center point of the microfluidic lane (VPC), and at the closest point to the media inlet (VPR) at the three main flow rates used in the experiment. Embryos are transported by the high flow rates in the serpentine channel, enabling gradual filling of all embryo incubators.


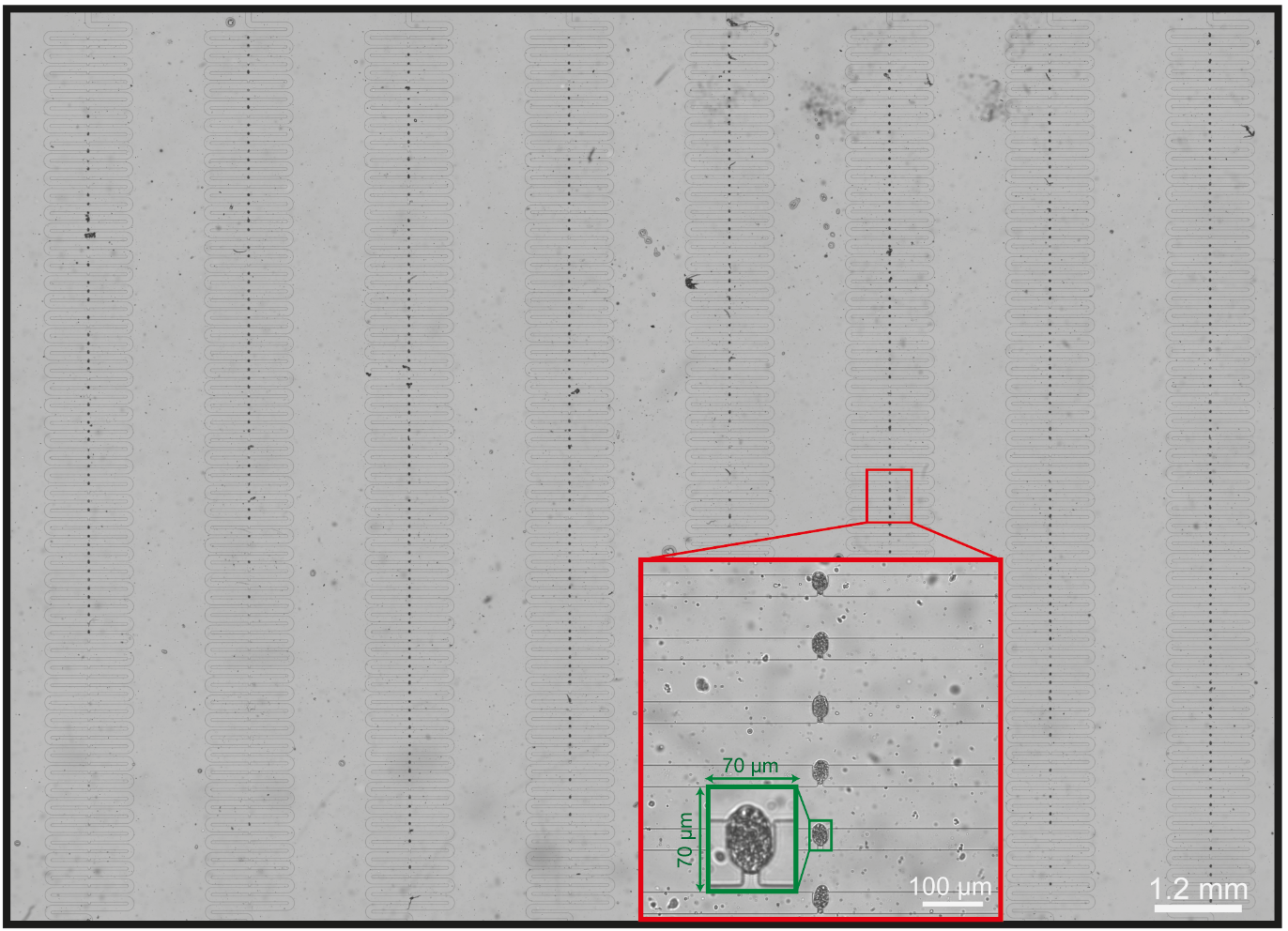


**Fig. S2. Stitched image covering 8 microfluidic lanes each of which having 100 embryo incubators.** Real-time images containing six embryo incubators (in red) are collected during time-lapse imaging and 70 µm by 70 µm image patches, centered around each individual embryo incubator (in green) are provided to the automated phenotyping script for feature extraction.


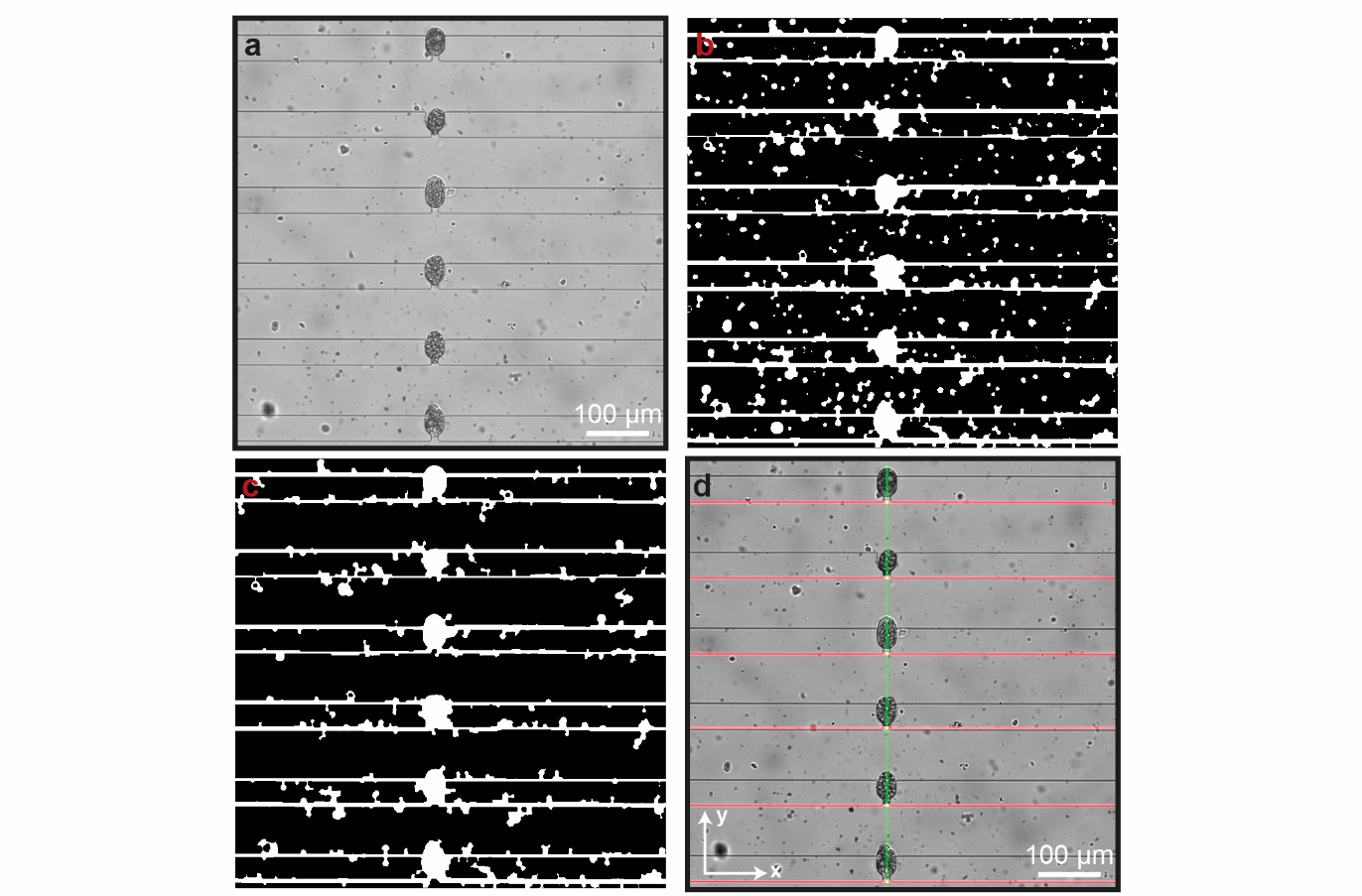


**Fig. S3.** **Details of the automated detection of the position of the embryo incubators**. **(a)** The first real-time brightfield image during time-lapse imaging was utilized. **(b)** Canny edge detector was run to reveal the edge map. We chose Canny edge detector ^1^ due to its ability of merging weak edges with nearby strong edges, leading to extraction of more robust edge information. **(c)** After edge detection, we applied a morphological closing operation on the resulting edge map to reveal all incubator boundaries without disruptions. Next, to find the y-coordinate corresponding to a horizontal boundary, we summed all pixels in a row of (c); and the resulting one-dimensional set of line-averaged pixel values allowed to allocate the horizontal PDMS boundaries (two for each incubator). **(d)** For each incubator, the lower PDMS boundary was selected (red line). A similar procedure was followed for finding the x-coordinate corresponding to the embryo incubators (green line). After revealing x- and y-coordinates of each embryo incubator for each position of our motorized stage, we cropped image patches of 70 µm × 70 µm (corresponding to 200 × 200 pixels) around each embryo incubator to be used for subsequent stages of our algorithm.


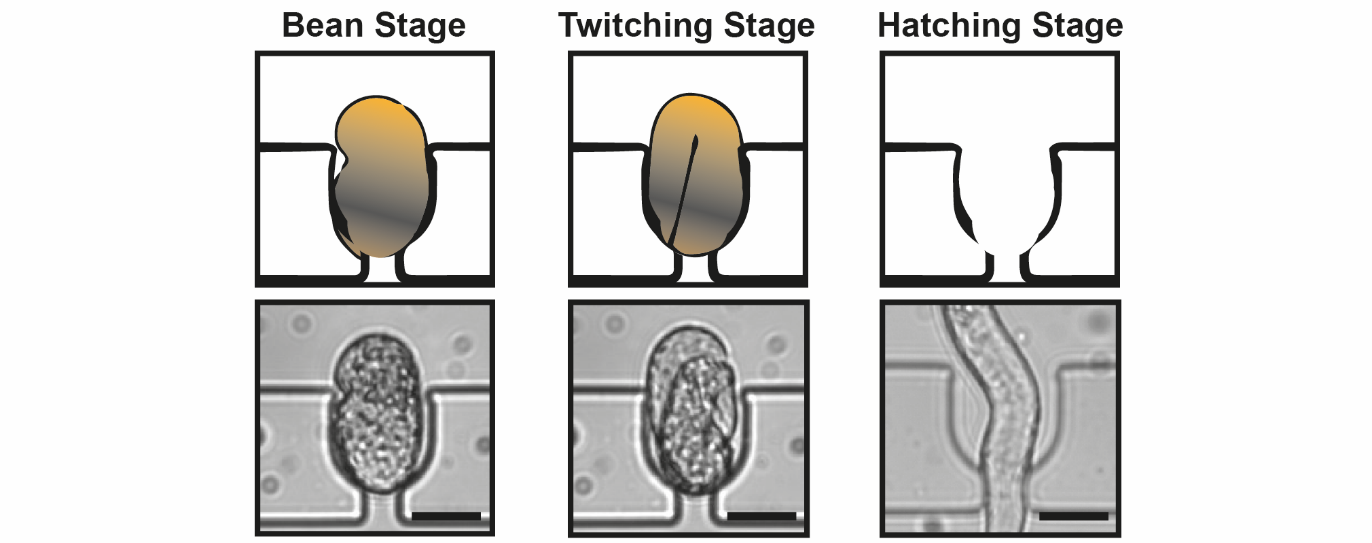


**Fig. S4.** **Schematic and real-time images of the bean, twitching and hatching stage transitions.** Scale bars: 20 µm.


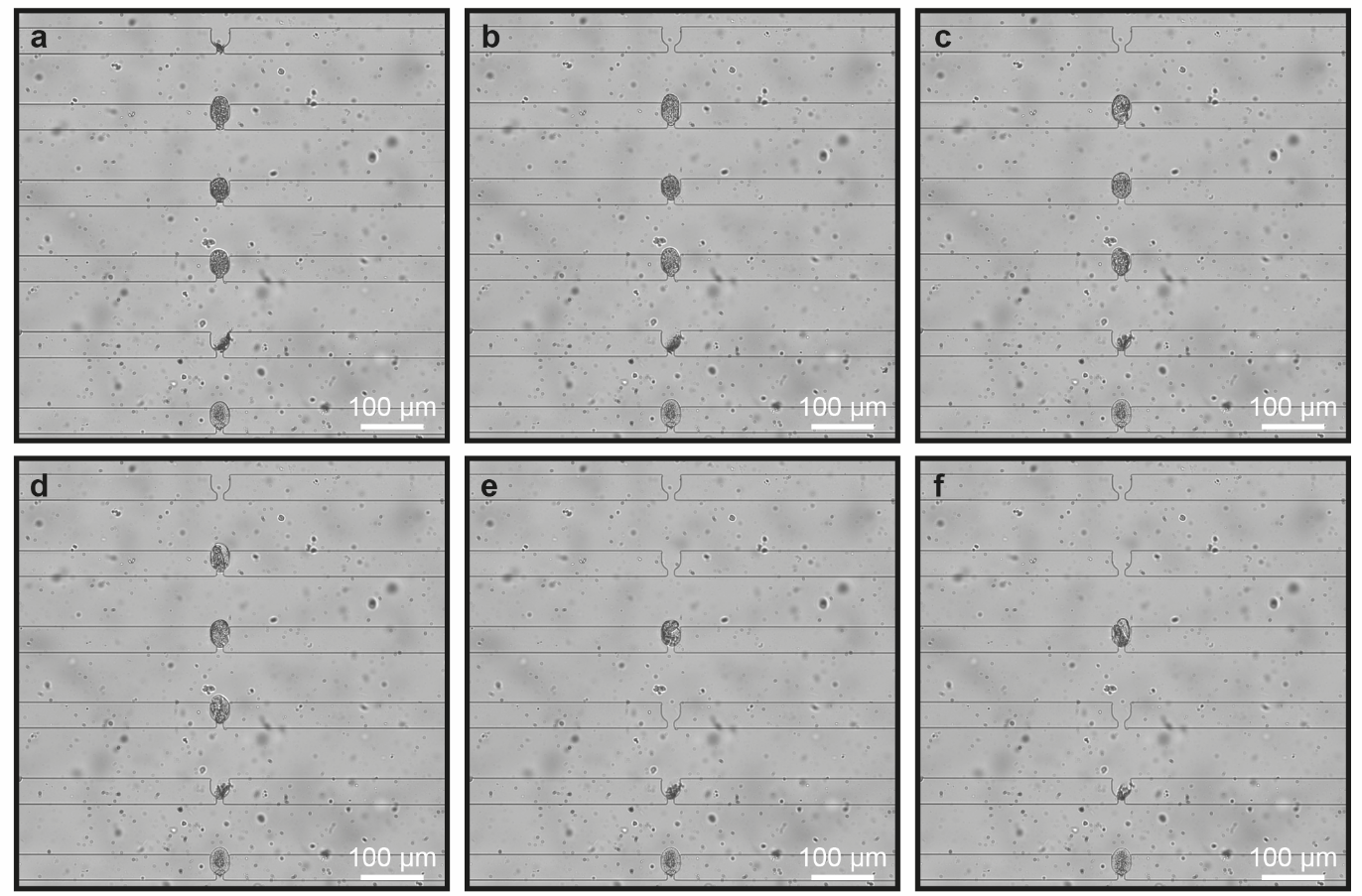


**Fig. S5.** **Real-time images of six embryo incubators in a timespan of 12 hours**. A real-time brightfield image in **(a)** the beginning, after **(b)** 145 minutes, **(c)** 290 minutes, **(d)** 435 minutes, **(e)** 580 minutes, and **(f)** 720 minutes of the experiment. From top to down, the algorithm categorized the embryos as; “Empty Embryo Incubator”, “Normal”, “Late Hatching”, “Unclear Stage” “Dead” and “Dead”. The “Normal” embryo had bean, twitching and hatching stage detections at 185, 270 and 565 minutes, respectively. The “Late Hatching” embryo had twitching stage detection at 440 minutes. The “Unclear Stage” embryo had twitching and hatching stage detections at 260 and 565 minutes, respectively. We noticed that the 5^th^ embryo incubator was classified as “Dead” even though the correct result was “Empty Embryo Incubator”. Likewise, corrected values for the bean stage of “Normal” embryo and the twitching stage of “Late Hatching” embryo were 210 and 465 minutes, respectively.


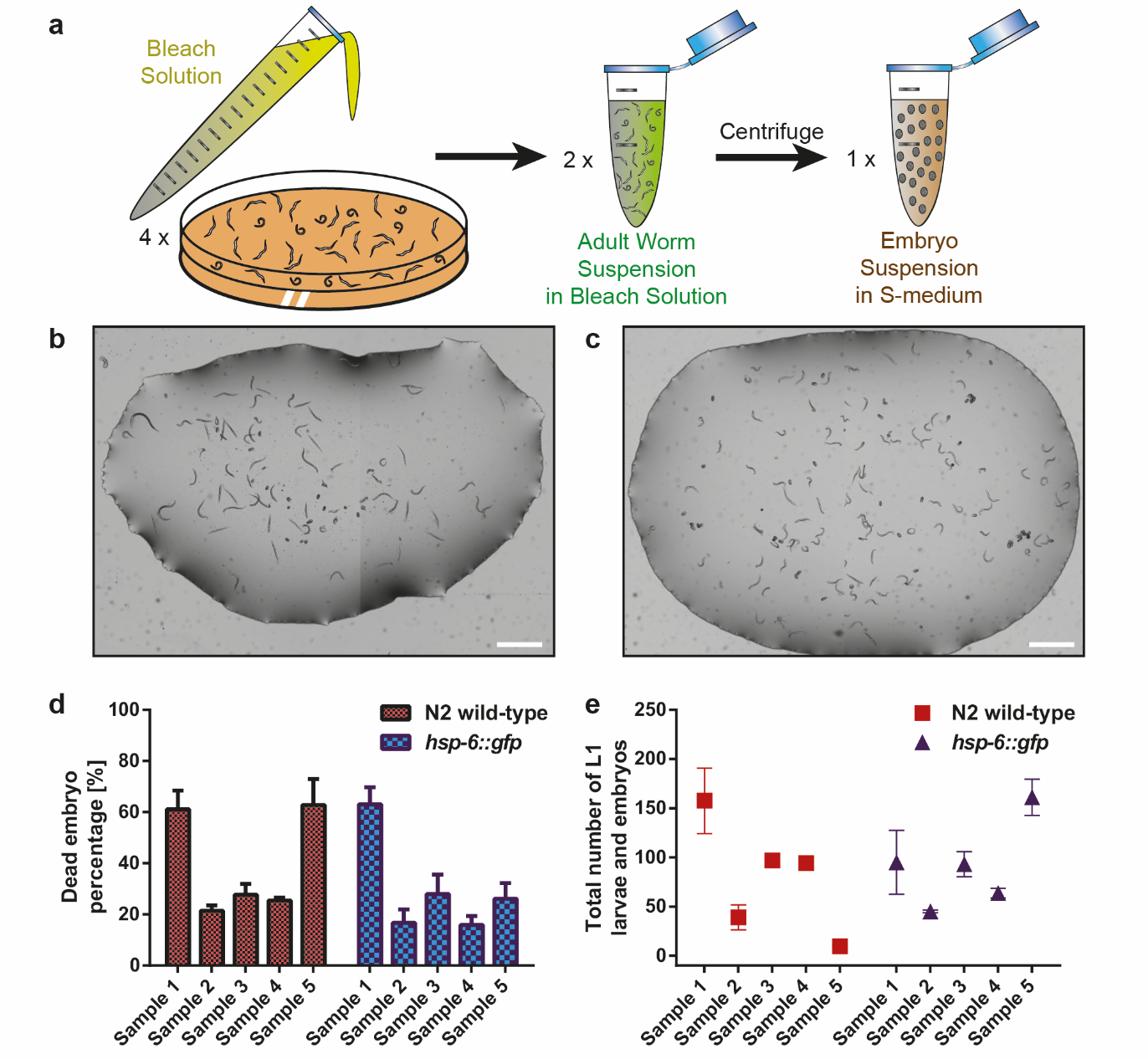


**Fig. S6. Bleaching procedure to obtain a massive amount of *C. elegans* embryos. (a)** **Schematic protocol.** 5 mL of bleach solution - 0.33 mL of 4 M sodium hydroxide, 3.66 mL of DI water and 1 mL of 7-10% sodium hypochlorite solution - is distributed on 4 gravid adult worm plates that contain 500-600 worms. The solution is suspended in two 1.5 mL Eppendorf tubes, and then centrifuged 4 times to replace bleach solution with S-medium. The single 1.5 mL Eppendorf tube containing all embryos is aliquotted in eight 500 µL Eppendorf tubes (one for use for each lane of the chip) and embryo loading is initiated. **Photographs of (b) N2 wild-type and (c) *hsp-6::gfp* worms and embryos in 2 µL samples obtained one day after the bleaching protocol.** Scale bars: 500 µm. Immediately after the bleaching protocol, only embryos keep their integrity. The larvae seen in (b) and (c) hence result from the hatching of embryos, confirming the viable status of the latter after bleaching. Dead embryos are still visible as such. **(d)** **Dead embryo percentage of five different samples for N2 wild-type and *hsp-6::gfp* strains.** Each sample was obtained via our bleaching procedure and images were acquired one day after – as shown in (b) and (c) – to analyze the dead embryo percentage. Variability can be associated to slightly varying local bleaching conditions on the agar plate. **(e) Total number of L1 larvae and embryos in the same 2 µL samples shown in (d).** Variability is an indicator of the variations in the initial gravid adult worm populations on the agar plate.


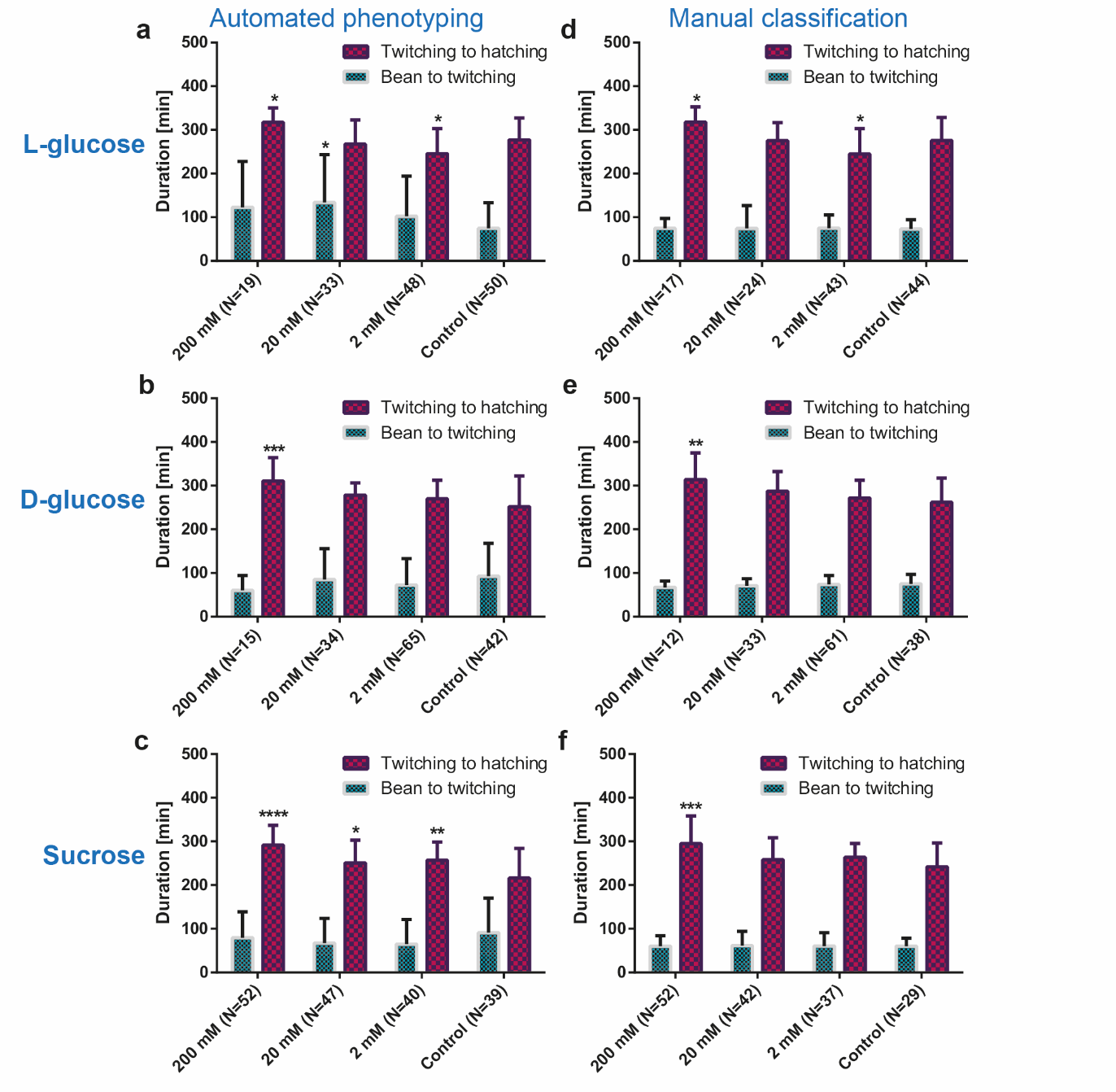


**Fig. S7.** **Results of our automated phenotyping script compared to the manually classified experimental results for the L-glucose, D-glucose and sucrose dose effect on the duration of the embryonic stages of “Normal” wild-type embryos**. **(a-c)** Automated phenotyping results of the embryo development duration for wild-type embryos when using (a) L-glucose, (b) D-glucose, and (c) sucrose are compared to **(d-f)** the corresponding manually classified results. Data are expressed as mean ± SEM, * p ≤ 0.05, ** p ≤ 0.01, *** p ≤ 0.001, **** p ≤ 0.0001. The number N is the total number of “Normal” embryos studied for a particular condition.


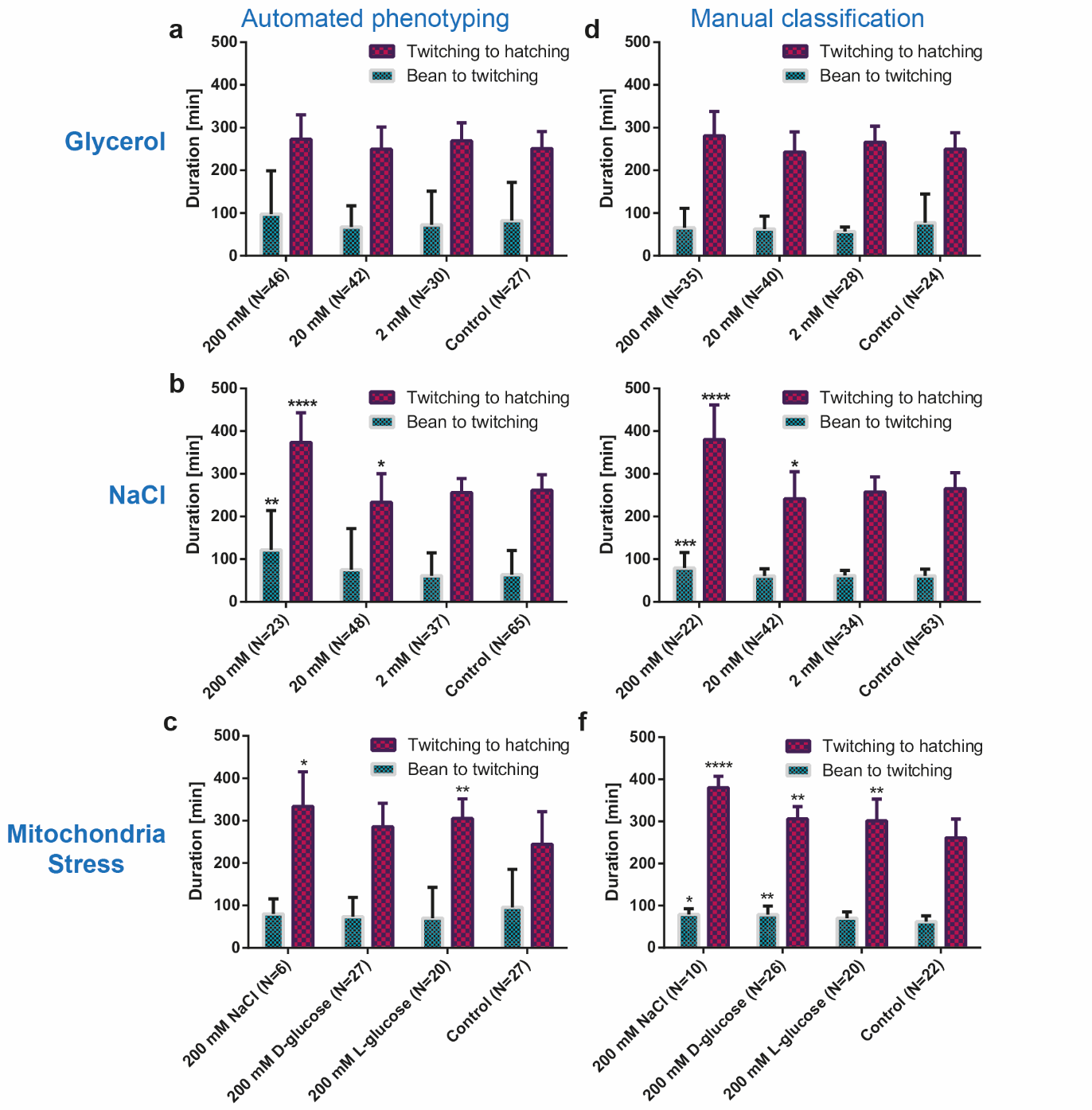


**Fig. S8.** **Results of our automated phenotyping script compared to the manually classified experimental results for the glycerol and NaCl dose effect on the duration of the embryonic stages of “Normal” wild-type embryos and of “Normal” *hsp-6::gfp* embryos under 200 mM NaCl, D-glucose and L-glucose**. **(a-c)** Automated phenotyping results of the embryo development duration for wild-type embryos when using (a) glycerol and (b) NaCl, and for *hsp-6::gfp* embryos when using (c) 200 mM NaCl, D-glucose, and L-glucose, compared to **(d-f)** the corresponding manually classified results. Data are expressed as mean ± SEM, * p ≤ 0.05, ** p ≤ 0.01, *** p ≤ 0.001, **** p ≤ 0.0001. The number N is the total number of “Normal” embryos studied for a particular condition.

**Table S1. Comparison of the automated phenotyping data to the manually classified data for the embryo states.** Percentage comparison of the automated phenotyping algorithm to our manually classification based on Dead, Unclear Stage, Empty Embryo Incubator, Late Hatching, Normal and Normal Embryo over Alive Embryo for 200, 20, 2 mM and the control (0 mM) conditions of L-glucose, D-glucose, sucrose, glycerol and NaCl of wild-type *C. elegans* embryos; and for 200 mM NaCl, 200 mM D-glucose, 200 mM L-glucose and the control condition (0 mM) of *hsp-6::gfp C. elegans* embryos.

| **Compound** | **Dose** | **Dead**  **Embryo [%]** | | **Unclear**  **Stage**  **Embryo**  **[%]** | | **Empty**  **Embryo**  **Incubator**  **[%]** | | **Late**  **Hatching**  **Embryo**  **[%]** | | | **Normal**  **Embryo**  **[%]** | | | | **Normal**  **Embryo Over Alive Embryo**  **[%]** | | |
| --- | --- | --- | --- | --- | --- | --- | --- | --- | --- | --- | --- | --- | --- | --- | --- | --- | --- |
| **(C)ode / (M)anual** | | **C** | **M** | **C** | **M** | **C** | **M** | **C** | **M** | | **C** | | **M** | | **C** | | **M** |
| **L-glucose** | 200 mM | 59.8 | 58.7 | 25.2 | 26.5 | 18.9 | 20.9 | 3.1 | 1.6 | | 12.0 | | 13.5 | | 29.7 | | 32.8 |
|  | 20 mM | 36.3 | 35 | 45.1 | 50.1 | 8.5 | 11.1 | 0.6 | 0 | | 18.1 | | 14.1 | | 28.4 | | 21.7 |
|  | 2 mM | 22.2 | 27.9 | 48.9 | 45.9 | 10 | 14 | 2.2 | 1.2 | | 26.7 | | 25 | | 34.3 | | 34.7 |
|  | Control | 38.5 | 42.3 | 30.5 | 30.4 | 13 | 16 | 2.3 | 0 | | 28.7 | | 27.4 | | 46.7 | | 47.4 |
| **D-glucose** | 200 mM | 56.1 | 56.6 | 26.2 | 27.7 | 18 | 20.5 | 8.5 | 8.2 | | 9.1 | | 7.5 | | 20.8 | | 17.4 |
|  | 20 mM | 52.7 | 54.2 | 22 | 21.8 | 25 | 29 | 2.7 | 0.7 | | 22.7 | | 23.2 | | 47.9 | | 50.8 |
|  | 2 mM | 37.2 | 44.4 | 22.4 | 18.9 | 8.5 | 15.5 | 4.9 | 0.6 | | 35.5 | | 36.1 | | 56.5 | | 64.9 |
|  | Control | 48 | 58.9 | 24 | 16.6 | 14.5 | 18.5 | 3.5 | 1.2 | | 24.6 | | 23.3 | | 47.2 | | 56.7 |
| **sucrose** | 200 mM | 19.4 | 22.3 | 51.1 | 48.6 | 10 | 10.5 | 0.6 | 0.6 | | 28.9 | | 28.5 | | 35.9 | | 36.7 |
|  | 20 mM | 25.7 | 27.1 | 45.7 | 47.6 | 12.1 | 17 | 1.7 | 0 | | 26.9 | | 25.3 | | 36.2 | | 34.7 |
|  | 2 mM | 27.4 | 29.8 | 49.1 | 48.5 | 10.7 | 14.5 | 0.6 | 0 | | 22.9 | | 21.6 | | 31.5 | | 30.8 |
|  | Control | 30.1 | 34.9 | 45.2 | 46.1 | 17 | 24 | 1.2 | 0 | | 23.5 | | 19.1 | | 33.6 | | 29.3 |
| **glycerol** | 200 mM | 37.1 | 34.4 | 38.1 | 46.7 | 1.5 | 2.5 | 1.5 | 1 | | 23.4 | | 18 | | 37.1 | | 27.3 |
|  | 20 mM | 29 | 35.5 | 48.7 | 43 | 3 | 7 | 0.5 | 0 | | 21.8 | | 21.5 | | 30.7 | | 33.3 |
|  | 2 mM | 44.9 | 46.9 | 35.3 | 35.6 | 16.5 | 20 | 1.8 | 0 | | 18.0 | | 17.5 | | 32.6 | | 32.9 |
|  | Control | 36.5 | 35.3 | 45.9 | 50.6 | 9 | 15 | 2.8 | 0 | | 14.9 | | 14.1 | | 23.5 | | 21.8 |
| **NaCl** | 200 mM | 42.8 | 33.6 | 13.3 | 19 | 10 | 10.5 | 31.1 | 35.2 | | 12.8 | | 12.3 | | 22.3 | | 18.5 |
|  | 20 mM | 32.6 | 40.5 | 37 | 34.8 | 8 | 11 | 4.3 | 1.1 | | 26.1 | | 23.6 | | 37.7 | | 39.6 |
|  | 2 mM | 29.3 | 30.8 | 42.7 | 43.4 | 25 | 28.5 | 3.3 | 2.1 | | 24.7 | | 23.8 | | 34.9 | | 34.3 |
|  | Control | 18.5 | 22.4 | 45.1 | 43.2 | 8 | 8.5 | 1.1 | 0 | 35.3 | | 34.4 | | 43.3 | | 44.4 | |
| **Stress**  **induction** | 200 mM  NaCl | 77.1 | 45.7 | 10 | 14.3 | 30 | 30 | 8.6 | 32.9 | 4.3 | | 7.1 | | 18.8 | | 13.2 | |
|  | 200 mM D-glucose | 51.6 | 38.3 | 29.3 | 42.9 | 21.1 | 23 | 1.9 | 1.9 | 17.2 | | 16.9 | | 35.5 | | 27.4 | |
|  | 200 mM  L-glucose | 52.4 | 43.9 | 27.4 | 31.7 | 38 | 38.5 | 4 | 8.1 | 16.1 | | 16.3 | | 33.9 | | 29 | |
|  | Control | 44.7 | 42.1 | 31.8 | 39.7 | 34 | 39.5 | 3 | 0 | 20.5 | | 18.2 | | 37.0 | | 31.4 | |

A “Dead” embryo detection problem occurred for 200 mM NaCl. At this dose, the algorithm classified some manually classified “Late Hatching” embryos as “Dead”. However, this is understandable, as unhatched embryos after 12 hours of culture may indicate indeed that the embryo or the first larval stages are dead.

**Table S2. Confusion matrix of the automated phenotyping algorithm for L-glucose with no error and with 5 frames error tolerance.** True Positive (TP), False Positive (FP), True Negative (TN), False Negative (FN), Sensitivity and Specificity results of the automated phenotyping script for 200, 20, 2 mM, control (0 mM) and the sum of all conditions of L-glucose of wild-type *C. elegans* embryos with no error tolerance (meaning that the automatic detection of an embryonic transition is considered as true, if it takes place in exactly the same time-lapse sequence frame as evaluated manually), and with 5 frames error tolerance (meaning that the automatic detection of an embryonic transition is considered as true, if it takes place in a frame of the time-lapse sequence that is not further than 5 frames apart (*i.e.* less than 25 min) from the frame in which it was evaluated manually).

| **L-glucose with no error tolerance** | **Detection** | **TP** | **FP** | **TN** | **FN** | **Sensitivity** | **Specificity** |
| --- | --- | --- | --- | --- | --- | --- | --- |
| **200 mM** | State | 187 |  |  | 9 | 95.4 % |  |
|  | Bean | 10 | 1 | 178 | 7 | 58.8 % | 99.4 % |
|  | Twitching | 57 | 3 | 132 | 4 | 93.4 % | 97.8 % |
|  | Hatching | 60 | 0 | 136 | 0 | 100 % | 100 % |
| **20 mM** | State | 173 |  |  | 26 | 86.9 % |  |
|  | Bean | 15 | 8 | 167 | 9 | 62.5 % | 95.4 % |
|  | Twitching | 102 | 6 | 83 | 8 | 92.7 % | 93.3 % |
|  | Hatching | 110 | 5 | 84 | 0 | 100 % | 94.4 % |
| **2 mM** | State | 174 |  |  | 26 | 87 % |  |
|  | Bean | 28 | 5 | 152 | 15 | 65.1 % | 96.8 % |
|  | Twitching | 106 | 16 | 62 | 16 | 86.9 % | 79.5 % |
|  | Hatching | 119 | 17 | 62 | 2 | 98.3 % | 78.5 % |
| **Control** | State | 168 |  |  | 32 | 84 % |  |
|  | Bean | 28 | 8 | 148 | 16 | 63.6 % | 94.9 % |
|  | Twitching | 76 | 20 | 93 | 11 | 87.4 % | 82.3 % |
|  | Hatching | 89 | 20 | 91 | 0 | 100 % | 82 % |
| **All** | State | 702 |  |  | 93 | 88.3 % |  |
|  | Bean | 81 | 22 | 645 | 47 | 63.3 % | 96.7 % |
|  | Twitching | 341 | 45 | 370 | 39 | 89.7 % | 89.2 % |
|  | Hatching | 378 | 42 | 373 | 2 | 99.5 % | 89.9 % |
|  | | | | | | | |
| **L-glucose with 5 frames tolerance** | **Detection** | **TP** | **FP** | **TN** | **FN** | **Sensitivity** | **Specificity** |
| **200 mM** | State | 187 |  |  | 9 | 95.4 % |  |
|  | Bean | 11 | 1 | 178 | 6 | 64.7 % | 99.4 % |
|  | Twitching | 59 | 3 | 132 | 2 | 96.7 % | 97.8 % |
|  | Hatching | 60 | 0 | 136 | 0 | 100 % | 100 % |
| **20 mM** | State | 173 |  |  | 26 | 86.9 % |  |
|  | Bean | 17 | 8 | 167 | 7 | 70.8 % | 95.4 % |
|  | Twitching | 106 | 6 | 83 | 4 | 96.4 % | 93.3 % |
|  | Hatching | 110 | 5 | 84 | 0 | 100 % | 94.4 % |
| **2 mM** | State | 174 |  |  | 26 | 87 % |  |
|  | Bean | 28 | 5 | 152 | 15 | 65.1 % | 96.8 % |
|  | Twitching | 111 | 16 | 62 | 11 | 91 % | 79.5 % |
|  | Hatching | 120 | 17 | 62 | 1 | 99.2 % | 78.5 % |
| **Control** | State | 168 |  |  | 32 | 84 % |  |
|  | Bean | 29 | 8 | 148 | 15 | 65.9 % | 94.9 % |
|  | Twitching | 77 | 20 | 93 | 10 | 88.5 % | 82.3 % |
|  | Hatching | 89 | 20 | 91 | 0 | 100 % | 82 % |
| **All** | State | 702 |  |  | 93 | 88.3 % |  |
|  | Bean | 85 | 26 | 645 | 39 | 66.4 % | 96.7 % |
|  | Twitching | 353 | 45 | 370 | 27 | 92.9 % | 89.2 % |
|  | Hatching | 379 | 42 | 373 | 1 | 99.7 % | 89.9 % |

**Table S3. Confusion matrix of the automated phenotyping algorithm for D-glucose with no error and with 5 frames error tolerance.** True Positive (TP), False Positive (FP), True Negative (TN), False Negative (FN), Sensitivity and Specificity results of the automated phenotyping script for 200, 20, 2 mM, control (0 mM) and the sum of all conditions of D-glucose of wild-type *C. elegans* embryos with no error tolerance (meaning that the automatic detection of an embryonic transition is considered as true, if it takes place in exactly the same time-lapse sequence frame as evaluated manually), and with 5 frames error tolerance (meaning that the automatic detection of an embryonic transition is considered as true, if it takes place in a frame of the time-lapse sequence that is not further than 5 frames apart (*i.e.* less than 25 min) from the frame in which it was evaluated manually).

| **D-glucose with no error tolerance** | **Detection** | **TP** | **FP** | **TN** | **FN** | **Sensitivity** | **Specificity** |
| --- | --- | --- | --- | --- | --- | --- | --- |
| **200 mM** | State | 187 |  |  | 13 | 93.5 % |  |
|  | Bean | 8 | 3 | 185 | 4 | 66.7 % | 98.4 % |
|  | Twitching | 60 | 6 | 127 | 7 | 89.6 % | 95.5 % |
|  | Hatching | 54 | 6 | 140 | 0 | 100 % | 95.9 % |
| **20 mM** | State | 178 |  |  | 22 | 89 % |  |
|  | Bean | 17 | 0 | 167 | 16 | 51.5 % | 100 % |
|  | Twitching | 57 | 13 | 126 | 4 | 93.4 % | 90.7 % |
|  | Hatching | 60 | 11 | 129 | 0 | 100 % | 92.1 % |
| **2 mM** | State | 170 |  |  | 30 | 85 % |  |
|  | Bean | 32 | 5 | 134 | 29 | 52.5 % | 96.4 % |
|  | Twitching | 80 | 23 | 86 | 11 | 87.9 % | 78.9 % |
|  | Hatching | 90 | 16 | 93 | 1 | 98.9 % | 85.3 % |
| **Control** | State | 170 |  |  | 30 | 85 % |  |
|  | Bean | 24 | 4 | 158 | 14 | 63.2 % | 97.5 % |
|  | Twitching | 51 | 26 | 111 | 12 | 81 % | 81 % |
|  | Hatching | 62 | 20 | 116 | 2 | 96.9 % | 85.3 % |
| **All** | State | 705 |  |  | 95 | 88.1 % |  |
|  | Bean | 81 | 12 | 644 | 63 | 56.3 % | 98.2 % |
|  | Twitching | 248 | 68 | 450 | 34 | 87.9 % | 86.9 % |
|  | Hatching | 266 | 53 | 478 | 3 | 98.9 % | 90 % |
|  | | | | | | | |
| **D-glucose with 5 frames tolerance** | **Detection** | **TP** | **FP** | **TN** | **FN** | **Sensitivity** | **Specificity** |
| **200 mM** | State | 187 |  |  | 13 | 93.5 % |  |
|  | Bean | 9 | 3 | 185 | 3 | 75 % | 98.4 % |
|  | Twitching | 64 | 6 | 127 | 3 | 95.5 % | 95.5 % |
|  | Hatching | 54 | 6 | 140 | 0 | 100 % | 95.9 % |
| **20 mM** | State | 178 |  |  | 22 | 89 % |  |
|  | Bean | 18 | 0 | 167 | 15 | 54.5 % | 100 % |
|  | Twitching | 60 | 13 | 126 | 1 | 98.4 % | 90.7 % |
|  | Hatching | 60 | 11 | 129 | 0 | 100 % | 92.1 % |
| **2 mM** | State | 170 |  |  | 30 | 85 % |  |
|  | Bean | 36 | 5 | 134 | 25 | 59 % | 96.4 % |
|  | Twitching | 85 | 23 | 86 | 6 | 93.4 % | 78.9 % |
|  | Hatching | 90 | 16 | 93 | 1 | 98.9 % | 85.3 % |
| **Control** | State | 170 |  |  | 30 | 85 % |  |
|  | Bean | 26 | 4 | 158 | 12 | 68.4 % | 97.5 % |
|  | Twitching | 56 | 26 | 111 | 7 | 88.9 % | 81 % |
|  | Hatching | 62 | 20 | 116 | 2 | 96.9 % | 85.3 % |
| **All** | State | 705 |  |  | 95 | 88.1 % |  |
|  | Bean | 89 | 12 | 644 | 55 | 61.8 % | 98.2 % |
|  | Twitching | 265 | 68 | 450 | 17 | 94 % | 86.9 % |
|  | Hatching | 266 | 53 | 478 | 3 | 98.9 % | 90 % |

**Table S4. Confusion matrix of the automated phenotyping algorithm for sucrose with no error and with 5 frames error tolerance.** True Positive (TP), False Positive (FP), True Negative (TN), False Negative (FN), Sensitivity and Specificity results of the automated phenotyping script for 200, 20, 2 mM, control (0 mM) and the sum of all conditions of sucrose of wild-type *C. elegans* embryos with no error tolerance (meaning that the automatic detection of an embryonic transition is considered as true, if it takes place in exactly the same time-lapse sequence frame as evaluated manually), and with 5 frames error tolerance (meaning that the automatic detection of an embryonic transition is considered as true, if it takes place in a frame of the time-lapse sequence that is not further than 5 frames apart (*i.e.* less than 25 min) from the frame in which it was evaluated manually).

| **sucrose with no error tolerance** | **Detection** | **TP** | **FP** | **TN** | **FN** | **Sensitivity** | **Specificity** |
| --- | --- | --- | --- | --- | --- | --- | --- |
| **200 mM** | State | 189 |  |  | 11 | 94.5 % |  |
|  | Bean | 25 | 0 | 148 | 27 | 48.1 % | 100 % |
|  | Twitching | 107 | 10 | 55 | 28 | 79.3 % | 84.6 % |
|  | Hatching | 130 | 10 | 58 | 2 | 98.5 % | 85.3 % |
| **20 mM** | State | 175 |  |  | 25 | 87.5 % |  |
|  | Bean | 31 | 6 | 152 | 11 | 73.8 % | 96.2 % |
|  | Twitching | 101 | 16 | 68 | 15 | 87.1 % | 81 % |
|  | Hatching | 117 | 11 | 70 | 2 | 98.3 % | 86.4 % |
| **2 mM** | State | 177 |  |  | 23 | 88.5 % |  |
|  | Bean | 21 | 7 | 156 | 16 | 56.8 % | 95.7 % |
|  | Twitching | 89 | 20 | 66 | 25 | 78.1 % | 76.7 % |
|  | Hatching | 111 | 17 | 68 | 4 | 96.5 % | 80 % |
| **Control** | State | 167 |  |  | 33 | 83.5 % |  |
|  | Bean | 19 | 10 | 161 | 10 | 65.5 % | 94.2 % |
|  | Twitching | 65 | 26 | 86 | 23 | 73.9 % | 76.8 % |
|  | Hatching | 92 | 20 | 84 | 4 | 95.8 % | 80.8 % |
| **All** | State | 708 |  |  | 92 | 88.5 % |  |
|  | Bean | 96 | 23 | 617 | 64 | 60 % | 96.4 % |
|  | Twitching | 362 | 72 | 275 | 91 | 79.9 % | 79.3 % |
|  | Hatching | 450 | 58 | 280 | 12 | 97.4 % | 82.9 % |
|  | | | | | | | |
| **sucrose with 5 frames tolerance** | **Detection** | **TP** | **FP** | **TN** | **FN** | **Sensitivity** | **Specificity** |
| **200 mM** | State | 189 |  |  | 11 | 94.5 % |  |
|  | Bean | 36 | 0 | 148 | 16 | 69.2 % | 100 % |
|  | Twitching | 113 | 10 | 55 | 22 | 83.7 % | 84.6 % |
|  | Hatching | 130 | 10 | 58 | 2 | 98.5 % | 85.3 % |
| **20 mM** | State | 175 |  |  | 25 | 87.5 % |  |
|  | Bean | 34 | 6 | 152 | 8 | 81 % | 96.2 % |
|  | Twitching | 111 | 16 | 68 | 5 | 95.7 % | 81 % |
|  | Hatching | 117 | 11 | 70 | 2 | 98.3 % | 86.4 % |
| **2 mM** | State | 177 |  |  | 23 | 88.5 % |  |
|  | Bean | 24 | 7 | 156 | 13 | 64.9 % | 95.7 % |
|  | Twitching | 96 | 20 | 66 | 18 | 84.2 % | 76.7 % |
|  | Hatching | 111 | 17 | 68 | 4 | 96.5 % | 80 % |
| **Control** | State | 167 |  |  | 33 | 83.5 % |  |
|  | Bean | 20 | 10 | 161 | 9 | 69 % | 94.2 % |
|  | Twitching | 68 | 26 | 86 | 20 | 77.3 % | 76.8 % |
|  | Hatching | 93 | 20 | 84 | 3 | 96.9 % | 80.8 % |
| **All** | State | 708 |  |  | 92 | 88.5 % |  |
|  | Bean | 114 | 23 | 617 | 46 | 71.3 % | 96.4 % |
|  | Twitching | 388 | 72 | 275 | 65 | 85.7 % | 79.3 % |
|  | Hatching | 451 | 58 | 280 | 11 | 97.6 % | 82.8 % |

**Table S5. Confusion matrix of the automated phenotyping algorithm for glycerol with no error and with 5 frames error tolerance.** True Positive (TP), False Positive (FP), True Negative (TN), False Negative (FN), Sensitivity and Specificity results of the automated phenotyping script for 200, 20, 2 mM, control (0 mM) and the sum of all conditions of glycerol of wild-type *C. elegans* embryos with no error tolerance (meaning that the automatic detection of an embryonic transition is considered as true, if it takes place in exactly the same time-lapse sequence frame as evaluated manually), and with 5 frames error tolerance (meaning that the automatic detection of an embryonic transition is considered as true, if it takes place in a frame of the time-lapse sequence that is not further than 5 frames apart (*i.e.* less than 25 min) from the frame in which it was evaluated manually).

| **glycerol with no error tolerance** | **Detection** | **TP** | **FP** | **TN** | **FN** | **Sensitivity** | **Specificity** |
| --- | --- | --- | --- | --- | --- | --- | --- |
| **200 mM** | State | 177 |  |  | 23 | 88.5 % |  |
|  | Bean | 15 | 11 | 154 | 20 | 42.9 % | 93.3 % |
|  | Twitching | 94 | 17 | 67 | 22 | 81 % | 79.8 % |
|  | Hatching | 113 | 14 | 70 | 3 | 97.4 % | 83.3 % |
| **20 mM** | State | 173 |  |  | 27 | 86.5 % |  |
|  | Bean | 25 | 3 | 157 | 15 | 62.5 % | 98.1 % |
|  | Twitching | 96 | 21 | 59 | 24 | 80 % | 73.8 % |
|  | Hatching | 111 | 21 | 61 | 7 | 94.1 % | 74.4 % |
| **2 mM** | State | 183 |  |  | 17 | 91.5 % |  |
|  | Bean | 14 | 2 | 170 | 14 | 50 % | 98.8 % |
|  | Twitching | 71 | 10 | 106 | 13 | 84.5 % | 91.4 % |
|  | Hatching | 84 | 7 | 109 | 0 | 100 % | 94 % |
| **Control** | State | 160 |  |  | 40 | 80 % |  |
|  | Bean | 11 | 3 | 173 | 13 | 45.8 % | 98.3 % |
|  | Twitching | 83 | 27 | 75 | 15 | 84.7 % | 73.5 % |
|  | Hatching | 94 | 27 | 76 | 3 | 96.9 % | 73.8 % |
| **All** | State | 693 |  |  | 107 | 86.6 % |  |
|  | Bean | 65 | 19 | 654 | 62 | 51.2 % | 97.2 % |
|  | Twitching | 344 | 75 | 307 | 74 | 82.3 % | 80.4 % |
|  | Hatching | 402 | 69 | 316 | 13 | 96.9 % | 82.1 % |
|  | | | | | | | |
| **glycerol with 5 frames tolerance** | **Detection** | **TP** | **FP** | **TN** | **FN** | **Sensitivity** | **Specificity** |
| **200 mM** | State | 177 |  |  | 23 | 88.5 % |  |
|  | Bean | 17 | 11 | 154 | 18 | 48.6 % | 93.3 % |
|  | Twitching | 107 | 17 | 67 | 9 | 92.2 % | 79.8 % |
|  | Hatching | 113 | 14 | 70 | 3 | 97.4 % | 83.3 % |
| **20 mM** | State | 173 |  |  | 27 | 86.5 % |  |
|  | Bean | 32 | 3 | 157 | 8 | 80 % | 98.1 % |
|  | Twitching | 111 | 21 | 59 | 9 | 92.5 % | 73.8 % |
|  | Hatching | 111 | 21 | 61 | 7 | 94.1 % | 74.4 % |
| **2 mM** | State | 183 |  |  | 17 | 91.5 % |  |
|  | Bean | 19 | 2 | 170 | 9 | 67.9 % | 98.8 % |
|  | Twitching | 78 | 10 | 106 | 6 | 92.9 % | 91.4 % |
|  | Hatching | 84 | 7 | 109 | 0 | 100 % | 94 % |
| **Control** | State | 160 |  |  | 40 | 80 % |  |
|  | Bean | 12 | 3 | 173 | 12 | 50 % | 98.3 % |
|  | Twitching | 87 | 27 | 75 | 11 | 88.8 % | 73.5 % |
|  | Hatching | 94 | 27 | 76 | 3 | 96.9 % | 73.8 % |
| **All** | State | 693 |  |  | 107 | 86.6 % |  |
|  | Bean | 80 | 19 | 654 | 47 | 63 % | 97.2 % |
|  | Twitching | 383 | 75 | 307 | 35 | 91.6 % | 80.4 % |
|  | Hatching | 402 | 69 | 316 | 13 | 96.9 % | 82.1 % |

**Table S6. Confusion matrix of the automated phenotyping algorithm for NaCl with no error and with 5 frames error tolerance.** True Positive (TP), False Positive (FP), True Negative (TN), False Negative (FN), Sensitivity and Specificity results of the automated phenotyping script for 200, 20, 2 mM, control (0 mM) and the sum of all conditions of NaCl of wild-type *C. elegans* embryos with no error tolerance (meaning that the automatic detection of an embryonic transition is considered as true, if it takes place in exactly the same time-lapse sequence frame as evaluated manually), and with 5 frames error tolerance (meaning that the automatic detection of an embryonic transition is considered as true, if it takes place in a frame of the time-lapse sequence that is not further than 5 frames apart (*i.e.* less than 25 min) from the frame in which it was evaluated manually)..

| **NaCl with no error tolerance** | **Detection** | **TP** | **FP** | **TN** | **FN** | **Sensitivity** | **Specificity** |
| --- | --- | --- | --- | --- | --- | --- | --- |
| **200 mM** | State | 175 |  |  | 25 | 87.5 % |  |
|  | Bean | 10 | 1 | 177 | 12 | 45.5 % | 99.4 % |
|  | Twitching | 78 | 25 | 76 | 21 | 78.8 % | 75.2 % |
|  | Hatching | 46 | 11 | 143 | 0 | 100 % | 92.9 % |
| **20 mM** | State | 172 |  |  | 28 | 86 % |  |
|  | Bean | 23 | 6 | 152 | 19 | 54.8 % | 96.2 % |
|  | Twitching | 89 | 22 | 74 | 15 | 85.6 % | 77.1 % |
|  | Hatching | 100 | 18 | 81 | 1 | 99 % | 81.8 % |
| **2 mM** | State | 182 |  |  | 18 | 91 % |  |
|  | Bean | 19 | 1 | 165 | 15 | 55.9 % | 99.4 % |
|  | Twitching | 82 | 11 | 92 | 15 | 84.53 % | 89.3 % |
|  | Hatching | 92 | 9 | 97 | 2 | 97.9 % | 91.5 % |
| **Control** | State | 185 |  |  | 15 | 92.5 % |  |
|  | Bean | 33 | 2 | 135 | 30 | 52.4 % | 98.5 % |
|  | Twitching | 120 | 12 | 48 | 20 | 85.7 % | 80 % |
|  | Hatching | 140 | 10 | 50 | 0 | 100 % | 83.3 % |
| **All** | State | 714 |  |  | 86 | 89.3 % |  |
|  | Bean | 85 | 10 | 629 | 76 | 52.8 % | 98.4 % |
|  | Twitching | 369 | 70 | 290 | 71 | 83.9 % | 80.6 % |
|  | Hatching | 378 | 48 | 371 | 3 | 99.2 % | 88.5 % |
|  | | | | | | | |
| **NaCl with 5 frames tolerance** | **Detection** | **TP** | **FP** | **TN** | **FN** | **Sensitivity** | **Specificity** |
| **200 mM** | State | 175 |  |  | 25 | 87.5 % |  |
|  | Bean | 12 | 1 | 177 | 10 | 54.5 % | 99.4 % |
|  | Twitching | 87 | 25 | 76 | 12 | 87.9 % | 75.2 % |
|  | Hatching | 46 | 11 | 143 | 0 | 100 % | 92.9 % |
| **20 mM** | State | 172 |  |  | 28 | 86 % |  |
|  | Bean | 28 | 6 | 152 | 14 | 66.7 % | 96.2 % |
|  | Twitching | 98 | 22 | 74 | 6 | 94.2 % | 77.1 % |
|  | Hatching | 100 | 18 | 81 | 1 | 99 % | 81.8 % |
| **2 mM** | State | 182 |  |  | 18 | 91 % |  |
|  | Bean | 22 | 1 | 165 | 12 | 64.7 % | 99.4 % |
|  | Twitching | 90 | 11 | 92 | 7 | 92.8 % | 89.3 % |
|  | Hatching | 92 | 9 | 97 | 2 | 97.9 % | 91.5 % |
| **Control** | State | 185 |  |  | 15 | 92.5 % |  |
|  | Bean | 39 | 2 | 135 | 24 | 61.9 % | 98.5 % |
|  | Twitching | 133 | 12 | 48 | 7 | 95 % | 80 % |
|  | Hatching | 140 | 10 | 50 | 0 | 100 % | 83.3 % |
| **All** | State | 714 |  |  | 86 | 89.3 % |  |
|  | Bean | 101 | 10 | 629 | 60 | 62.7 % | 98.4 % |
|  | Twitching | 408 | 70 | 290 | 32 | 92.7 % | 80.6 % |
|  | Hatching | 378 | 48 | 371 | 3 | 99.2 % | 88.5 % |

**Table S7. Confusion matrix of the automated phenotyping algorithm for *hsp-6::gfp* embryos with no error and with 5 frames error tolerance.** True Positive (TP), False Positive (FP), True Negative (TN), False Negative (FN), Sensitivity and Specificity results of the automated phenotyping script for 200 mM NaCl, 200 mM D-glucose, 200 mM L-glucose, control (0 mM) and the sum of all conditions of *hsp-6::gfp* *C. elegans* embryos with no error tolerance (meaning that the automatic detection of an embryonic transition is considered as true, if it takes place in exactly the same time-lapse sequence frame as evaluated manually), and with 5 frames error tolerance (meaning that the automatic detection of an embryonic transition is considered as true, if it takes place in a frame of the time-lapse sequence that is not further than 5 frames apart (*i.e.* less than 50 min) from the frame in which it was evaluated manually).

| ***hsp-6::gfp* with no error tolerance** | **Detection** | **TP** | **FP** | **TN** | **FN** | **Sensitivity** | **Specificity** |
| --- | --- | --- | --- | --- | --- | --- | --- |
| **200 mM NaCl** | State | 143 |  |  | 57 | 71.5 % |  |
|  | Bean | 4 | 2 | 190 | 4 | 50 % | 99 % |
|  | Twitching | 22 | 58 | 117 | 3 | 88 % | 66.9 % |
|  | Hatching | 16 | 13 | 170 | 1 | 94.1 % | 92.9 % |
| **200 mM D-glucose** | State | 173 |  |  | 27 | 86.5 % |  |
|  | Bean | 13 | 3 | 171 | 13 | 50 % | 98.3 % |
|  | Twitching | 68 | 24 | 103 | 5 | 93.2 % | 81.1 % |
|  | Hatching | 72 | 22 | 106 | 0 | 100 % | 82.8 % |
| **200 mM L-glucose** | State | 181 |  |  | 19 | 90.5 % |  |
|  | Bean | 7 | 0 | 180 | 13 | 35 % | 100 % |
|  | Twitching | 46 | 18 | 127 | 9 | 83.6 % | 87.6 % |
|  | Hatching | 51 | 11 | 138 | 0 | 100 % | 92.6 % |
| **Control** | State | 175 |  |  | 25 | 87.5 % |  |
|  | Bean | 17 | 5 | 173 | 5 | 77.3 % | 97.2 % |
|  | Twitching | 57 | 12 | 123 | 8 | 87.7 % | 91.1 % |
|  | Hatching | 63 | 11 | 125 | 1 | 98.4 % | 91.9 % |
| **All** | State | 672 |  |  | 128 | 84 % |  |
|  | Bean | 41 | 10 | 714 | 35 | 53.9 % | 98.6 % |
|  | Twitching | 193 | 112 | 470 | 25 | 88.5 % | 80.8 % |
|  | Hatching | 202 | 57 | 539 | 2 | 99 % | 90.4 % |
|  | | | | | | | |
| ***hsp-6::gfp* with 5 frames tolerance** | **Detection** | **TP** | **FP** | **TN** | **FN** | **Sensitivity** | **Specificity** |
| **200 mM NaCl** | State | 143 |  |  | 57 | 71.5 % |  |
|  | Bean | 4 | 2 | 190 | 4 | 50 % | 99 % |
|  | Twitching | 22 | 58 | 117 | 3 | 88 % | 66.9 % |
|  | Hatching | 16 | 13 | 170 | 1 | 94.1 % | 92.9 % |
| **200 mM D-glucose** | State | 173 |  |  | 27 | 86.5 % |  |
|  | Bean | 21 | 3 | 171 | 5 | 80.8 % | 98.3 % |
|  | Twitching | 72 | 24 | 103 | 1 | 98.6 % | 81.1 % |
|  | Hatching | 72 | 22 | 106 | 0 | 100 % | 82.8 % |
| **200 mM L-glucose** | State | 181 |  |  | 19 | 90.5 % |  |
|  | Bean | 14 | 0 | 180 | 6 | 70 % | 100 % |
|  | Twitching | 53 | 18 | 127 | 2 | 96.4 % | 87.6 % |
|  | Hatching | 51 | 11 | 138 | 0 | 100 % | 92.6 % |
| **Control** | State | 175 |  |  | 25 | 87.5 % |  |
|  | Bean | 20 | 5 | 173 | 2 | 90.9 % | 97.2 % |
|  | Twitching | 60 | 12 | 123 | 5 | 92.3 % | 91.1 % |
|  | Hatching | 63 | 11 | 125 | 1 | 98.4 % | 91.9 % |
| **All** | State | 672 |  |  | 128 | 84 % |  |
|  | Bean | 59 | 10 | 714 | 17 | 77.6 % | 98.6 % |
|  | Twitching | 207 | 112 | 470 | 11 | 95 % | 80.8 % |
|  | Hatching | 202 | 57 | 539 | 2 | 99 % | 90.4 % |


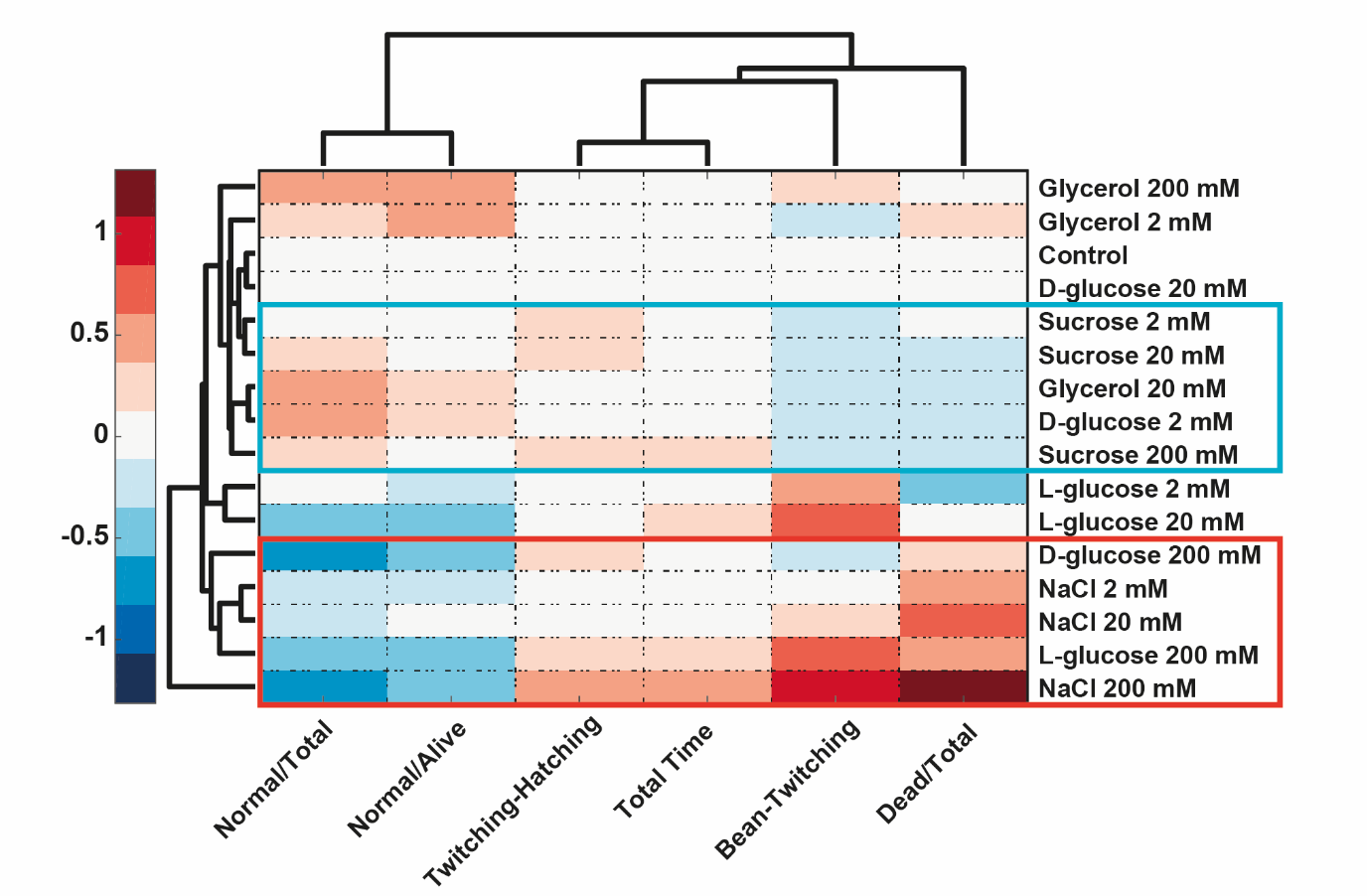


**Fig. S9.** **Study of the effect of various chemical compounds with different molarities on the development of wild-type *C. elegans* embryos.** The ratio of the number of “Normal” embryos over the total number of embryos placed in the incubators, the ratio of the number of “Normal” embryos over the number of total alive embryos, the twitching-to-hatching time interval, the total development time, the bean-to-twitching stage duration, and the number of “Dead” embryos over total embryos placed in the incubators were utilized as the phenotypic parameters. Zones in the clustergram that correspond to conditions that are in favor or disfavor of longevity are marked with blue and red rectangles, respectively. All measurements were based on N= 15 to 65 embryos.

In order to create a clustergram plot, the data was centered and normalized to allow for the comparison of datasets spread over different scales and orders of magnitude. In this case, only the magnitude of the relative deviation from the control results is taken into account. In particular, we used the mean value of each parameter of interest corresponding to a certain phenotype and normalized it by the corresponding value of the control condition with the following formula to result in the so-called normalized and centered parameter of interest *I_NCPOI_*:

|  | $I_{NCPOI}=\frac{I_{PI}}{I_{CCP}}-1$ |  |
| --- | --- | --- |

Here, *I_PI_* and *I_CCP_* are the original parameter of interest and the corresponding control parameter, respectively.

Our clustergram also reports two more parameters that we did not display previously (parameters “Normal/Total” and “Total Time”). The “Normal” embryo over all embryos ratio was included in addition to the “Normal” embryo over alive embryo ratio. In fact, we did not notice significant variation in these two parameters. The other parameter was the total development time, which was calculated as the average bean-to-twitching development time plus the average twitching-to-hatching development time. From this clustergram, we noticed that 2, 20 and 200 mM sucrose, 20 mM glycerol and 2 mM D-glucose concentrations revealed a positive stimulus on the health of embryos (in the blue rectangle). In these conditions, the “Dead” embryo percentage was reduced and the “Normal” embryo observation percentage was increased. In parallel, there was not a significant variation in the twitching-to-hatching and total development times. While 200 mM glycerol concentration did not have a significant impact on embryos, 200 mM of L-glucose, D-glucose and NaCl showed toxic effects, thereby reducing the “Normal” embryo percentage, increasing the “Dead” embryo rate and the development time (in the red rectangle). More specifically, NaCl as an ionic compound severely impacted embryos at all molarities. We presumed that ionic compounds could penetrate inside the eggshell as the bleaching step weakens the outer embryo shell layers ^2,3^. The thus weakened eggshell allowed ionic compounds dissolved in S-medium buffer to easily pass inside the egg and influence the embryo development. NaCl also alters the ionic strength of the buffer, thus creating a gradient across the embryo membrane and worsening the water loss induced by the hyperosmotic environment. By observing the dendrograms of the clustergram, NaCl at 200 mM concentration appeared to be isolated from the rest of the conditions and hence, had the most pronounced influence.


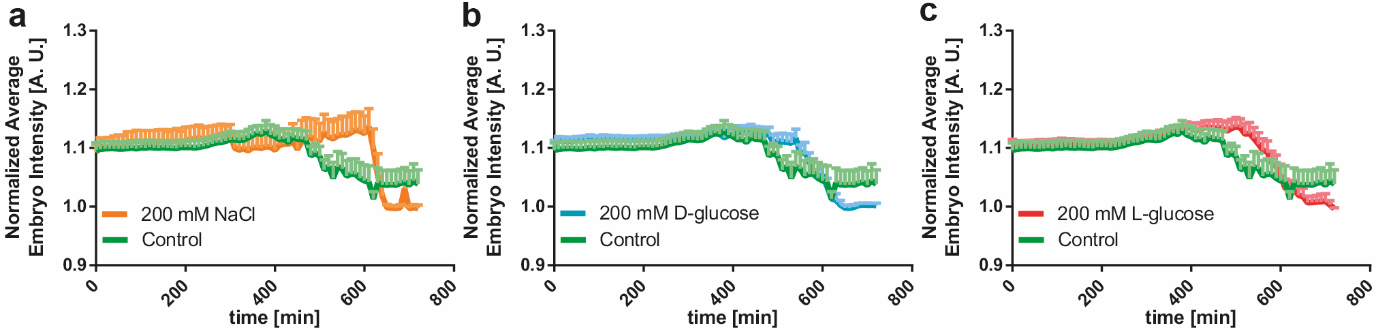


**Fig. S10.** **Averaged fluorescent intensity as a function of time during exposure to various chemical compounds at 200 mM of “Normal” *hsp-6::gfp* *C. elegans* embryos**. We observe mitochondrial stress induction in these embryos, as evidenced by an increase of the background-corrected fluorescence for **(a)** 200 mM NaCl, **(b)** 200 mM D-glucose and **(c)** 200 mM L-glucose compared to the control (0 mM) condition. The drop that is observed in the curves after 400 minutes corresponds to the onset of embryo hatching events, lowering the average intensity results due to the significantly reduced intensity contributions from embryo-free incubators.


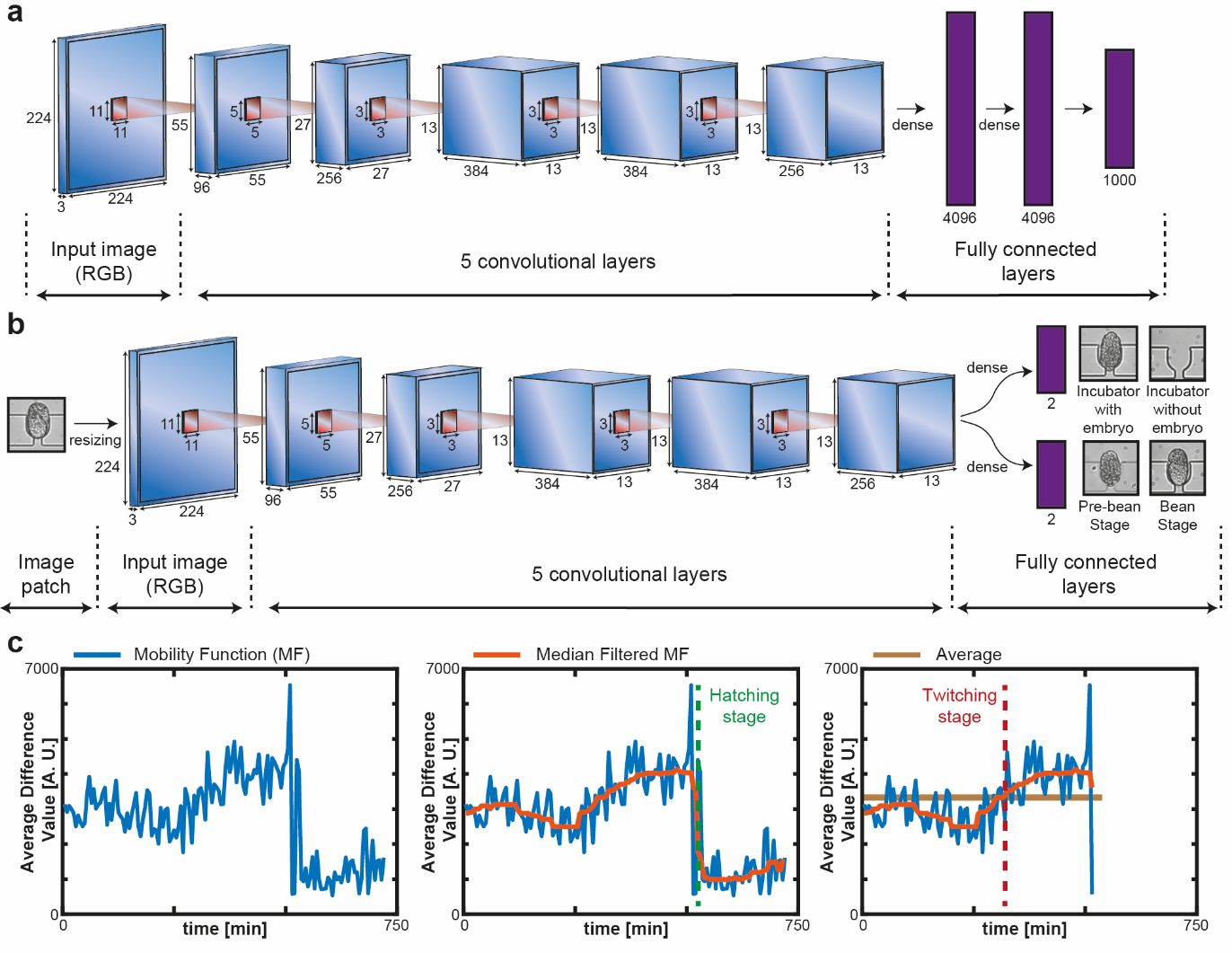


**Fig. S11.** **Schematic representation of the algorithms used for the automated phenotyping of *C. elegans* embryos. (a)** **Original AlexNet architecture.** AlexNet is one of the most well-known Convolutional Neural Network (CNN) architectures ^4^. First, a sample image is, resized to 224×224×3 pixels (the factor 3 represents the 3 color channels). The resized image is filtered through five convolutional layers to extract features from it. Basically, a convolution layer is used to divide the input image into small tiles, on which a convolution operation is applied (multiplying the pixel intensity values of the tile with those of a small matrix window that represents a predefined feature and is slided over the tile). The tiles obtained from the input image that are used in the five subsequent convolution layers are convolved with matrix windows with different weights of the matrix elements, also known as Kernels, and having sizes of 11×11, 5×5, 3×3, 3×3 and 3×3 pixels, respectively. After each convolution operation, a feature map with the most significant features that are actually present in the original image is created, such as edges, blobs, shapes, etc.. Moreover, after each convolution, a rectified linear unit (ReLU) is used to set all negative values in the feature map to zero. Then, only the largest values in the feature map are stored, also known as a Max Pooling operation, meaning that the size of the feature map is reduced by down-sampling. After the convolutional layers, three so-called fully connected layers perform a classification based on the features extracted by the former. The fully connected layers generate a probability for each of the classification options the model is trying to predict. Initially, during training of the network, known images are fed to the network and all learnable parameters of the fully connected layers are changed such that the network at the end gives the desired classification results. **(b) Our specific architecture used for embryo classification, based on AlexNet.** We used AlexNet convolutional layers while we removed the fully connected layers. Instead, we used two fully connected layer branches; one to determine whether an incubator contains an embryo or not and another to detect if the embryo is either in the pre-bean or bean stage of embryogenesis. Outputs of both branches are two-dimensional vectors, where the value of each field represents the probability of its associated status. **(c)** **Automatic detection of the onset of the twitching stage of an embryo.** (left) The differences of the intensity values of all pixels present in two consecutive embryo image patches of a time-lapse sequence are averaged, representing the mobility function (MF) of the embryo. (center) The MF is median-filtered. (right) Only the median-filtered MF before the hatching time is considered and averaged. The time point, at which this Average function intersects with the median-filtered MF defines the start of the twitching stage.

**References**

1 J. Canny, in *IEEE Transactions on Pattern Analysis and Machine Intelligence*, 1986, vol. 8, pp. 679–698.

2 A. Carvalho, S. K. Olson, E. Gutierrez, K. Zhang, L. B. Noble, E. Zanin, A. Desai, A. Groisman and K. Oegema, *PLoS One*, 2011, **6**, e24656.

3 L. G. Edgar, *Methods Cell Biol.*, 1995, **48**, 303–321.

4 A. Krizhevsky, I. Sutskever and G. E. Hinton, in *Proceedings of Advances In Neural Information Processing Systems 25*, 2012, pp. 1090–1098.

**Supplementary Movies**

**Movie S1.** Real-time video during the initial embryo loading

**Movie S2.** Real-time scan of the multiplexed lanes after the finalization of the initial embryo loading

**Movie S3.** Stack of time-lapse brightfield images of a single position with three embryos in six incubators, corresponding to Fig. S5
